# Current Research and Statistical Practices in Sport Science and a Need for Change

**DOI:** 10.3390/sports5040087

**Published:** 2017-11-15

**Authors:** Jake R. Bernards, Kimitake Sato, G. Gregory Haff, Caleb D. Bazyler

**Affiliations:** 1 Center of Excellence for Sport Science and Coach Education, Department of Sport, Exercise, Recreation, and Kinesiology, East Tennessee State University, Johnson City, TN 37614, USA; satok1@etsu.edu (K.S.); bazyler@etsu.edu (C.D.B.); 2 Center for Exercise and Sport Science Research, Edith Cowan University, Joondalup, WA 6027, Australia; g.haff@ecu.edu.au

**Keywords:** sport science, statistics, inference, magnitude, Bayesian

## Abstract

Current research ideologies in sport science allow for the possibility of investigators producing statistically significant results to help fit the outcome into a predetermined theory. Additionally, under the current Neyman-Pearson statistical structure, some argue that null hypothesis significant testing (NHST) under the frequentist approach is flawed, regardless. For example, a p-value is unable to measure the probability that the studied hypothesis is true, unable to measure the size of an effect or the importance of a result, and unable to provide a good measure of evidence regarding a model or hypothesis. Many of these downfalls are key questions researchers strive to answer following an investigation. Therefore, a shift towards a magnitude-based inference model, and eventually a fully Bayesian framework, is thought to be a better fit from a statistical standpoint and may be an improved way to address biases within the literature. The goal of this article is to shed light on the current research and statistical shortcomings the field of sport science faces today, and offer potential solutions to help guide future research practices.

## 1. The Problem

Although the common goal of many researchers remains the same, the validity of the sport science body of literature may be in question because of common research practices and the current statistical framework applied [1,2,3]. Recently, journals are beginning to address existing research and statistical practices through journal wide initiatives to increase the reproducibility of results found in the literature. While such initiatives act as a first step, a shift in the current statistical framework may be a better solution to ensure the field of sport science continually progresses. While pressure to increase the volume of publications in the academic setting progresses, it has become increasingly tempting to deviate from the scientific framework that has been proven to be crucial for discovery; rigor, reproducibility, and transparency [4]. Although the ambition to develop novel and innovative findings remains the primary goal of the current academic infrastructure, the outcome can become biased and unchallenged [5]. The occurrence of biased and unchallenged outcomes in sport science can be attributed to at least two factors.

First, the current academic/publication structure to only reward new, unique and ground-breaking findings instils a need for producing research that has statistically significant results. Second, existing statistical practices lend themselves to nearly any result, to be interpreted at the author’s discretion. With biased interpretations of nearly any statistical result, a more transparent statistical structure could help lead the reader to make claims based off the results, rather than the authors’ interpretations of such results [1,6]. This is very different from the current dogma of *p* < 0.05, therefore the intervention works. Therefore, the goal of this article is to shed light on the potential problems the field of sport science faces, and to offer solutions to help guide future research practices. 

A persistent problem in current research practice involves the multitude of ways an individual can manipulate their data to produce statistically significant results in the absence of a true effect. Examples include manipulation of statistics to produce statistically significant results (*p*-hacking) and hypothesis after results are known (HARKing) [7,8]. Such practices make it is nearly impossible for the reader to know which findings are a discovery and which are produced. While the production of statistically significant results may be advantageous for the researcher in the short-term, it can be detrimental to the literature in the long-term. When non-statistical data is manipulated to produce a significant *p*-value, it appears to the reader of the investigation that there is likely an effect of the treatment. It is plausible that a future reader will be interested in expanding on the topic, even though the effect of the treatment may have been unsuccessful, but only appeared to be effective following manipulation of the data. Repeat this process over and over, and the body of literature can venture down research avenues that are based off an original study that had no true effect to begin with. A similar concept was the underlining focus of Thomas Kuhn’s essay, *The Structure of Scientific Revolutions* [9]. If researchers meld their data to fit individual theories through *p*-hacking and HARKing, individual theories will persist and the field of sport science is likely to progress at a much slower rate. 

A secondary struggle that has stemmed from current research practice includes the “*file-drawer effect*”. By striving to primarily publish the latest findings within the field, journals have likely created a biased body of literature for coaches and investigators to pull from as a result of the “*file-drawer effect*” [10]. Due to the “*file-drawer effect*”, common strength and conditioning practices that are “*evidence based*” may appear to be effective simply because a handful of studies showed statistical significance; however, similar studies may have revealed no significance and never made it to publication. This “*file-drawer effect*” causes two key problems in the body of literature. First, this system can often cause researchers to undertake unwarranted research by basing their hypotheses, theories, and future experiments on a study that may have no effect but was shown as statistically significant by *p*-hacking or HARKing. Second, by primarily including studies that show statistical significance and not including unpublished, non-significant studies, the body of literature becomes biased and, therefore, the common practice of performing a meta-analysis is likely to also be biased. 

Furthermore, under null hypothesis significance testing (NHST), *p* < 0.05 is sufficient to state an intervention was effective without any regard to the magnitude of the effect. This shortcoming allows researchers to dredge their data looking for any relationship that will lead to a statistically significant finding. This can lead to an inflated rate of Type I errors that may go unnoticed with the lack of a replication process. Beyond the strategies to meld the data into the *p* < 0.05 box, some argue that the frequentist approach of NHST is flawed, regardless [11,12,13,14].

For example, when a hypothesis is not specified prior to data collection and analysis, the widely used multiway ANOVA exhibits a multiple comparisons issue [11]. As Cramer et al. [11] point out, when a 2 × 3 ANOVA is computed with a “*let’s see what we can find*” approach, the probability of making a minimum of one Type I error (familywise error rate) inflates to 0.14 or 14% as opposed to the thought error rate set to five-percent when a multiple correction adjustment is omitted. Currently, it is only taken on faith that the author had a hypothesis prior to the analysis process. 

Cumming [12] argues that NHST prompts researchers to see the world as black or white, and to formulate research aims to make conclusions in absolute terms—an effect is statistically significant or it is not; it exists or it does not. However, rarely is our field of sports science black and white. Furthermore, the sole use of *p*-values shifts investigators’ focus away from the practicality of a finding to simply claim statistical significance without providing a detailed description of what may have occurred. 

From a statistical standpoint, a *p*-value cannot; (1) measure the probability that the studied hypothesis is true, (2) measure the size of an effect or the importance of a result, or (3) provide a good measure of evidence regarding a model or hypothesis [1,15]. Moreover, decisions about an effect based on some “magic threshold” may be biased, regardless of how the threshold is defined [13,16]. Lastly, *p*-values are sample-size dependent, a major limitation in the field of sport science if the study is done with an elite athletic population. For example, a strength program may show significance with a sample of 12, yet with two dropouts may miss the effect [14]. Therefore, an alternative research and statistical model may better suit our field. 

## 2. The Solution

In sports science, there are two primary study designs: hypothesis generating (exploratory) and hypothesis testing (experimental). While both types of studies are central components of the applied research model typically used in sport science [17], it is important that there is a clear understanding of their roles in research and when the appropriate design is necessitated. As Tukey (1997) stated, if we do not explore, we might miss valuable insights that could suggest new research directions. This statement resonates within our field, where the smallest variances can be the difference in medalling or going home empty handed. However, in an exploratory analysis of the data, results must be clearly identified as speculative, and warrant further investigation with a developed hypothesis revealed during the analysis, a key concept that is often forgotten [18].

When planning a study, one proposed model sports scientists can follow is the Applied Research Model for Sport Science (ARMSS) [17]. The model incorporates both exploratory and experimental study designs linked together in a sequential manner to maximize the transferability of the research to a sport setting. ARMSS is an eight-stage model that includes;

(1) Defining the problem
(2) Descriptive research (hypothesis generating)
(3) Predictors of performance
(4) Experimental testing of predictors
(5) Determinants of key performance predictors
(6) Efficacy studies (controlled laboratory or field)
(7) Barriers to uptake
(8) Implementation studies (real sporting setting)

Once a problem has been defined, an investigator can then begin exploratory research to determine relationships that specific variables may have to the problem. This process helps to provide investigators domains of where to look for potential solutions. Following an exploratory finding, results need to be verified via replication to ensure an effect is in fact present. However, because scientific journals tend to favour novel findings, this crucial step of replication rarely occurs [17]. Therefore, during subsequent novel studies on the same topic, researchers should also attempt to replicate previous findings within their investigations by including previously identified correlations alongside the novel aspects [17].

The process of replication can then repeat itself to continually progress a topic forward while also presenting novel findings to advance the investigators academic career. Ideally, a replication study that is built with this framework will keep key features of the original investigation while modifying others to give a converging perspective. This method will not only increase the confidence in the original finding but will also begin to explore additional variables that may influence it [12]. Once a relationship between key variables and a specific problem have been determined, the researchers can then proceed to more traditional research designs that help determine causal relationship between the variables and a problem [17]. Once a causal relationship has been determined, the efficacy of specific investigations addressing the problem can be investigated. Finally, for the transfer and adoption of research outcomes to be effective, evidence must show that the use of the innovation is both feasible and effective in practice. This can be accomplished by evaluating the findings in a sport setting to ensure it is an improvement to current practice [17]. However, considering the flaws of NHST, novel statistical approaches are needed to support the ARMSS model.

## 3. Current Alternative Statistical Methods

Detailed in this section are common alternative statistics, and a brief explanation of how to conduct/interpret them.

### 3.1. Smallest Worthwhile Change

A key struggle of studying elite athletics, whether it is for research purposes or to determine if a training program is moving an individual in a meaningful direction, is sample size. Under the current statistical model of NHST, it is often impossible to achieve a statistically significant *p*-value due to extremely small samples and considerably small effects. However, the smallest of effects can be the sole difference when you are dealing with athletes of the highest calibre. Two metrics can be utilized in determining the smallest difference that can lead to a meaningful change in performance; smallest worthwhile change and smallest real difference. 

The smallest worthwhile change, also termed smallest meaningful change and smallest clinically important difference is calculated one of two ways, dependent on the nature of the sport. For team sports, the smallest worthwhile change can be calculated as; 0.2 × between-subject SD [19,20]. For a variable to be considered capable of detecting the smallest worthwhile change under this formula, the typical error of the measurement must be less than the smallest worthwhile change. 

When calculating the smallest worthwhile change in individual sports, you must first determine the sport-specific coefficient of variation, whether in the literature or through your own research. After obtaining this value, 0.3 of the coefficient of variation equates to a top-tiered athlete medaling once for every ten races when racing equally matched elite athletes [21,22]. A value of 0.3 was determined via simulation, and equates to a top-tiered athlete gaining one extra medal every 10 races performed. This same technique was used to determine Hopkins’ guidelines for interpreting effect sizes. Values of 0.9, 1.6, 2.5, and 4.0 of a CV give an extra 3, 5, 7, and 9 medals per 10 races, respectively. When assessing a fitness test in a team setting, rather than using the coefficient of variation, one should use the standardized change (Cohen’s *d_z_*), as there is no clear relationship between fitness-test performance and team performance [23]. The smallest worthwhile change is then equal to 0.2 of *d* [23].

The smallest real differences, also termed the smallest detectable difference, is the smallest measurement change that can be interpreted as a real difference beyond zero [24]. Calculation of this metric can be computed as:

(1) $$1.96\times \sqrt{2}\times SEM$$

where *SEM* equals the standard error of measurement [25]. Because measurement error causes the observed measurement to differ from the individual’s true value, an error band can be calculated to express the uncertainty of the difference between the two observed scores. When the smallest real difference error band contains zero, the difference between the two measurements may have been induced by error alone, and may not be a result of the intervention [25]. 

### 3.2. Comparing Correlations 

A common question in sports science is to compare two correlations obtained from a single sample of subjects, often a team, between multiple predictor variables and a single common dependent variable. Much like *d* can compare results measured in various units, the *r* to z-transformation allows an investigator to compare the correlation coefficients between a dependent variable and a set of independent variables. The first step to comparing multiple correlation coefficients to a dependent variable is to perform Fisher’s z-transformation, defined as:

(2) $$z=\frac{1}{2}\ln\left(\frac{1+r}{1-r}\right)$$

where ln is the natural log and *r* is the correlation of the two variables. Fisher’s z-transformation of sample correlation coefficients improves the normality substantially, especially for small sample sizes [26]. Following the transformation from *r* to *z*, the two correlated correlations can then be compared to determine what predictors can do a better job in predicting the variable in question [26]. This method can also be used when testing whether correlations with a common variable follow the pattern of magnitudes that a theory would predict [26].

Correlations that have been calculated from different samples can also be tested against one another. Comparing correlations from two samples can help determine if there is a significant difference in the correlations of two groups. For example, imagine you are collecting data on training age and jump height from men and women. The two resulting correlations can be tested against each other to determine if there is a significant difference in the correlation of both groups. To compare correlations from independent samples, we must first convert the coefficients to *z*-scores than can determine the *z*-score difference as:

(3) $$z_{Difference=\frac{z_{r_{1}}-z_{r_{2}}}{\sqrt{\frac{1}{N_{1}-3}+\frac{1}{\sqrt{N_{2}-3}}}}}$$

This score can then be used to determine the one- or two-tailed probabilities to determine significance [27]. 

### 3.3. Effect Size

Cohen’s *d* effect size is a *z*-score that will take the difference of two group means and divide the result by a standardizer if the assumption of homogeneity of variance is met. While there are multiple calculations used to determine *d*, the standardizer chosen is dependent on the study design [28]. For example, *d_s_* is calculated by using the pooled standard deviation of the groups and is used when investigating independent groups. When determining the effect in a one sample group, the standard deviation difference in scores can be used as the standardizer to calculate *d_z_*. When dealing with a small sample size and meta analyses, a Hedges’ *g* correction can be computed. Calculating Cohen’s *d_s_* based off sample averages may give a biased estimate of the population effect size, especially for samples under twenty participants [29]. Cohen’s *d_s_* can be converted to the adjusted Hedge’s *g_s_* by [28]:

(4) $$g_{s}=d_{s}\times \left(1-\frac{3}{4\left(n_{1}+n_{2}\right)-9}\right)$$

As outlined by Cohen (1988), *d* is also a metric of the magnitude of the effect with guidelines originally set forth by both Cohen and later updated specifically for sport science by Hopkins [30], and for resistance training studies by Rhea [31], to guide investigators toward interpreting the magnitude of the effect. As it currently stands, the guidelines set forth by Cohen Hopkins, and Rhea are detailed in Table 1. 

### 3.4. Confidence Intervals

The confidence interval (CI) can be defined as, “the likely range of the true, real, or population value of the statistic” within a given probability [22]. This range of values is unique in that, rather than there being a set probability of the CI containing the true population value of a given statistic, the CI will include the population’s true value a given number of times when replicated indefinitely. For example, if a study was replicated an indefinite number of times, 95% of the calculated CIs would include the population’s true value of its accompanying statistic, while 5% of the calculated CIs will not. Although ambiguous alone, the use of confidence intervals in combination with an effect size can help to show the precision of the effect size estimate. 

Confidence intervals can be computed for a wide range of commonly calculated statistics. Examples of metrics that often include a confidence interval in the literature are group means, mean difference, and effect size. When used in conjunction with an effect size, confidence intervals can be especially useful in making magnitude-based inferences.

### 3.5. Magnitude-Based Inferences

Pioneered by Dr. Hopkins and colleges in the early 2000s, making inferences from magnitude-based metrics can be accomplished by using a multi-level scale [22]. To do so, one can inspect the magnitudes covered by its effect size confidence interval and infer to what degree of the true value it could be [22]. While metrics such as a confidence interval may be vague when used alone, the confidence interval in conjunction with another statistic like effect size and smallest worthwhile change can take the typical polar reject-nonreject decision and transform it into a 3-level scale of magnitude (beneficial, trivial, and harmful) that inferences can be based off [22]. Inferences stemming from the 3-level scale of magnitudes result in “beneficial”, “trivial”, “harmful”, or “unclear”, dependent on the statistic/resulting confidence interval, a much more useful approach than the current “the effect is not statistically significant” response [22]. Incorporating such a method also has the added benefit of opening transparency and allowing the reader to determine their own inferences based on the results. Magnitude-based inferences can be made more accurate and informative by qualifying them with probabilities that help to reflect the uncertainty in the true value [33]. The qualitative probabilistic terms can be assigned using the scale put forth by Hopkins (2007); <0.5%, most unlikely or almost certainly not; 0.5–5%, very unlikely; 5–25%, unlikely or probably not; 25–75%, possibly; 75–95%, likely or probably; 95–99.5%, very likely; >99.5%, most likely or almost certainly [34].

### 3.6. Counter-Argument against Magnitude-Based Inferences

There are three common limitations of the magnitude-based inference model that advocates acknowledge. These include:

(1) A defined *a priori* with both a magnitude of the smallest important effect and the thresholds used to qualify likelihoods is needed [14].
(2) The investigator is invited to include his/her bias into the final interpretation [22].
(3) The potential of inflating the inferential error rate in increased [35,36].

The need for a strongly defined *a priori* should not be looked at as a limitation, but rather an advantage over the current NHST system. Rather than simply testing against the likelihood that all groups came from the same population, a defined *a priori* necessitates that the investigator adopts a conscious process when analysing and interpreting their data [14]. By simply testing a set of data against the NULL, you cannot strengthen a theory, but simply say that it effects the population in some way. There is no direction or strength to the claim. By defining *a priori*, the magnitude-based inference model can strengthen a theory by testing directly against itself. You are no longer testing against the void of the NULL, but against tangible expectations. The result is either a stronger or weaker than theory, dependent on the results. Moreover, the magnitude of the change can also help the degree to which the theory is strengthened or weakened.

The claim that magnitude-based inference increases the bias of a decision from the researcher does not hold substance as bias has already seeped into science under the current NHST framework. With publications allowing interpretations such as, “*weakly significant*” (*p* = 0.11), “*approaching formal significance*” (*p* = 0.1052), and “*not significantly … but clinically meaningful*” (*p* = 0.072) the NHST already allows for bias [37]. All the previous examples were pulled from published peer-reviewed journal articles with α set to 0.05. Under the current statistical framework, some investigators mold their findings to tell their own story. From the *p*-value alone, the reader cannot detect the practical significance or the magnitude of the differences.

Finally, the thought that magnitude-based inference increases the potential of inflating the inferential error rate has been shown not to be true. In a study conducted by Hopkins & Batterham (2016), results from 500,000 simulated randomized controlled trials, magnitude-based inference methods outperformed NHST in respect of inferential error rates. In addition, magnitude based inference also outperformed in terms of rates of publishable outcomes with suboptimal sample sizes and publication bias with such samples [35].

## 4. Bayesian Estimation

While the shift towards a magnitude-based inference model may act as a better fit for inference in sport science, a commitment towards a fully Bayesian model may act as a better solution for small effects and small sample sizes [38]. Criticism towards the magnitude inference model claim that a fully Bayesian approach may be a better solution [38,39].

In a study performed by Mengersen et al. [38], a fully Bayesian approach was shown to provide more direct probabilistic comparisons of treatments and able to identify small effects of interest, even with small sample sizes. Conclusions based off the Bayesian model were consistent with a magnitude-based inference approach and was determined to be a simple and effective way of analysing small effects while providing a rich set of results that are straightforward to interpret in terms of probabilistic statements [38].

In Mengersen’s comparison of statistical models, the authors applied a Bayesian model with a traditional statistical model to Humberstone-Gough et al. [40] study determining the effects of three training regimens; “Live High Train Low”, “Intermittent Hypoxic Exposure”, and “Placebo” on running performance and blood characteristics [40]. Results from the Bayesian model were consistent with those reported from the original investigation. However, the Bayesian approach allowed for a much more direct probabilistic interpretation of credible intervals and posterior probabilities [38].

Small effects on competitive performance are vital in elite athletics, and highly relevant for coaches and sport scientists when understanding the likely benefit or harm of a training program, recovery intervention, or any other facet surrounding the preparation process [38]. Adopting a Bayesian approach may by one approach to providing an answer. For example, in Mengersen’s Bayesian model of the altitude training data collected by Humberstone-Gough et al. [40], running economy improved by ~0.17 L (4.2%) more in the live high train low group when compared to intermittent hypoxic exposure. Data from the Bayesian model resulted in a 95% credible interval of −0.9 to −7.5% with a probability of ~0.99 that the true decrease in submaximal oxygen consumption is substantial (worthwhile) [38]. This is just one example of how a Bayesian model can allow for a much more detailed result, enabling the reader to gain better insight to the analysis. 

Bayesian methods are different from frequentist approaches in that the parameters are treated as random variables that have a true, but unknown value. These values are described by a posterior probability distribution that reflects the uncertainty associated with how well they are known based on the data [38]. The posterior distribution is calculated by:

Likelihood × prior

The likelihood describes the probability of observing the data given specified values of the parameters. The prior encapsulates beliefs about the probability of obtaining those independently of the data. Priors may either be developed using a range of information including previous experiments, historical data, and/or expert opinions, or priors can be uninformative to allow inferences to be driven by the observed data alone [38]. Priors allow the model to explore the consequences of beginning with varying information. Still, there can be many reasonable choices when defining the prior, all of which produce the same inference [41]. Even in the absence of previous knowledge, there is usually enough information to determine a plausible range of values that can be encoded directly into the prior and discount the plausibility of some parameter values (i.e., the negative associations between height and weight) [41].

It is important to note that there has been push back against adopting a Bayesian statistical model for various reasons. Historically, one of the biggest criticisms of Bayesian inference revolves around the prior [42]. While priors can be used to help produce results, much like *p*-hacking, it becomes increasingly obvious to the reader if done. Both Bayesian and non-Bayesian models are equally harried and dependent on likelihood functions and conventionalized model forms [41]. Therefore, if nothing more, adopting a Bayesian model is advantageous in both transparency and providing information for the reader to determine their own decision. 

A major drawback to adopting a fully Bayesian approach is that most current sport scientists are not trained in Bayesian methods. This is largely due to the progression of Bayesian approaches only recently becoming commonplace following advancements with the computer. However, the unique hurdles that continue to persist in sport science may lend itself to adopting a fully Bayesian approach [38]. Furthermore, decision making can also be enhanced by the richer probabilistic and inferential capability afforded by the Bayesian analysis [38]. For example, “the outcome of an intervention to improve athletic performance may be classified as ‘possible’ in some cases (acceptable probability of improving performance within minimal adverse effects) and hence lead to a decision of using, whereas in another context it may be deemed too risky (unacceptable risk of impairing performance due to specified risks) and lead to no action” [38]. In practice, these decisions may not coincide with the traditional statement of statistically significant effect at the alpha 0.05 level [43]. The intricacies of Bayesian inference go far beyond the extremely brief introduction provided here. Additional papers have been added to the supplemental section to act as a starting point to begin to make an individual decision in regards to adopting a Bayesian Model. Additionally, as with any cultural shift, many fields of science are currently debating if a Bayesian model is superior with strong arguments from both sides [38,42]. It is important to note that no one model can address every issue that occurs in science. It is the responsibility of the investigator to be as transparent and informative as possible to inform the reader of the results, something a Bayesian framework may be able to offer.

## 5. Conclusions

The practice of focusing exclusively on a dichotomous reject-nonreject decision strategy of NHST to determine the effect a treatment has on an outcome can impede scientific progress. [44] The data should lead us, instead of fitting the data to our hypotheses. One way to help ensure the practice of data manipulation does not have a place in our field is to adopt a more transparent and informative statistical model. In doing so, the author can provide a more detailed statistical analysis of what occurred in the investigation and the reader is able to make an individualized interpretation of the effectiveness of an intervention. The inclusion of confidence intervals, effect statistics, and other descriptive metrics to accompany the *p*-value under the NHST model is an easy and effective first step an investigator can make to produce more transparent research that is also more informative. However, a shift towards a magnitude-based inference model, and eventually a fully Bayesian approach, may be a better fit from a statistical standpoint, a reproducibility standpoint, and may be an improved way to address biases within the literature. All while being a superior model to deal with smaller samples sizes and small effects, two fundamental struggles in the field.

## Figures and Tables

**Table 1:** Effect size interpretation guidelines.

	Trivial	Small	Moderate	Large	Very Large	Nearly Perfect
Cohen [32]	N/A	0.1	0.3	0.5	N/A	N/A
Hopkins [30]	0–0.2	0.2–0.6	0.6–1.2	1.2–2.0	2.0–4.0	>4.0–∞
Rhea [31]: Untrained	<0.5	0.5–1.25	1.25–1.9	>2.0	N/A	N/A
Rhea [31]: Recreationally Trained	<0.35	0.35–0.8	0.8–1.5	>1.5	N/A	N/A
Rhea [31]: Highly Trained	<0.25	0.25–0.5	0.5–1.0	>1.0	N/A	N/A

## Supplementary Materials

The following are available online at www.mdpi.com/2075-4663/5/4/87/s1, Bayesian Versus Orthodox Statistics: Which Side Are You On?
