# Supplementary material for: Current Research and Statistical Practices in Sport Science and a Need for Change
Source: Sports (Basel). 2017 Nov 15;5(4):87. doi: 10.3390/sports5040087 (PMC5969020; doi:10.3390/sports5040087)
Supplement: Supplementary file 1 [file sports-05-00087-s001.pdf]

ASSOCIATION FOR
PSYCHOLOGICAL SCIENCE

Perspectives on Psychological Science
6(3) 274–290
© The Author(s) 2011
Reprints and permission:
sagepub.com/journalsPermissions.nav
DOI: 10.1177/1745691611406920
http://pps.sagepub.com
SAGE

Bayesian Versus Orthodox Statistics: Which Side Are You On?

Zoltan Dienes
School of Psychology, University of Sussex, Brighton, United Kingdom

Abstract

Researchers are often confused about what can be inferred from significance tests. One problem occurs when people apply Bayesian intuitions to significance testing—two approaches that must be firmly separated. This article presents some common situations in which the approaches come to different conclusions; you can see where your intuitions initially lie. The situations include multiple testing, deciding when to stop running participants, and when a theory was thought of relative to finding out results. The interpretation of nonsignificant results has also been persistently problematic in a way that Bayesian inference can clarify. The Bayesian and orthodox approaches are placed in the context of different notions of rationality, and I accuse myself and others as having been irrational in the way we have been using statistics on a key notion of rationality. The reader is shown how to apply Bayesian inference in practice, using free online software, to allow more coherent inferences from data.

Keywords

statistical inference, Bayes, significance testing, evidence, likelihood principle

Psychology and other disciplines have benefited enormously from having a rigorous procedure for extracting inferences from data. The question this article raises is whether we could be doing it better. Two main approaches are contrasted: orthodox statistics versus the Bayesian approach. Around the 1940s, the heated debate between the two camps was momentarily won in terms of what users of statistics did: Users followed the approach systematized by Jerzy Neyman and Egon Pearson (at least this approach defined norms; in practice, researchers often followed the somewhat different advice of Ronald Fisher; see e.g., Gigerenzer, 2004). But it was not that the intellectual debate was decisively won. It was more a practical matter of which approach had the most well worked-out applications at the time and which approach was conceptually easier for the researcher to apply. But now the practical problems have been largely solved; there is little to stop researchers from using the Bayesian approach in almost all circumstances. Thus the intellectual debate can be opened up again, and indeed it has (e.g., Baguley, in press; Hoijtink, Klugkist, & Boelen, 2008; Howard, Maxwell, & Fleming, 2000; Johansson, in press; Kruschke, 2010a, 2010b, 2010c, 2011, this issue; Rouder, Morey, Speckman, & Pratte, 2007; Rouder, Speckman, Sun, Morey, & Iverson, 2009; Taper & Lele, 2004; Wetzels et al., 2011, this issue). It is time for researchers to consider foundational issues in inference. And it is time to consider whether it is really advantageous that it takes less thought to calculate canned *p* values or whether it has led us astray in interpreting data (e.g., Harlow, Mulaik, & Steiger, 1997; Meehl, 1967; Royall, 1997; Ziliak & McCloskey, 2008), despite the benefits it has also provided. Indeed, I argue we would be most rational, under one intuitively compelling notion of rationality, to be Bayesians.

Test Your Intuitions

To see where your sympathies lie, at least initially, consider the three scenarios in Box 1, where the approaches give different responses. You might reject all the responses for a given scenario or feel attracted to more than one. Real research questions do not have pat answers, but see if, nonetheless, you have clear preferences. Almost all responses are consistent either with some statistical approach or with what a large section of researchers do in practice, so do not worry about picking out the one "right" response (though, given certain assumptions, I will argue that there is a right response).

Throughout the rest of this article, I will consider how to think about these scenarios. First, I review the basics of orthodox hypothesis testing to show how the starting assumptions of

Corresponding Author:
Zoltan Dienes, School of Psychology, University of Sussex, Brighton, BN1 9QH United Kingdom
E-mail: dienes@sussex.ac.uk

Box 1. Testing Your Intuitions in Three Research Scenarios

1. Stopping rule

You have run the 20 subjects you planned and have obtained a p *value of .08. Despite predicting a difference, you know this won't be convincing to any editor and run 20 more subjects. SPSS now gives a* p *of .01. Would you:*

a) Submit the study with all 40 participants and report an overall p of .01?

b) Regard the study as nonsignificant at the 5% level and stop pursuing the effect in question, as each individual 20-subject study had a p of .08?

c) Use a method of evaluating evidence that is not sensitive to your intentions concerning when you planned to stop collecting subjects, and base conclusions on all the data?

2. Planned versus post hoc

After collecting data in a three-way design, you find an unexpected partial two-way interaction, specifically you obtain a two-way interaction ($p = .03$*) for just the males and not the females. After talking to some colleagues and reading the literature, you realize there is a neat way of accounting for these results: Certain theories can be used to predict the interaction for the males but they say nothing about females. Would you:*

a) Write up the introduction based on the theories leading to a planned contrast for the males, which is then significant?

b) Treat the partial two-way as nonsignificant, as the three-way interaction was not significant, and the partial interaction won't survive corrections for post hoc testing?

c) Determine how strong the evidence of the partial two-way interaction is for the theory you put together to explain it, with no regard to whether you happen to think of the theory before seeing the data or afterwards, as all sorts of arbitrary factors could influence when you thought of a theory?

3. Multiple testing

You explore five possible ways of inducing subliminal perception as measured with priming. Each method interferes with vision in a different way. The test for each method has a power of 80% for a 5% significance level to detect the size of priming produced by conscious perception. Of these methods, the results for four are nonsignificant and one, Continuous Flash Suppression, is significant, $p = .03$*, with a priming effect numerically similar in size to that found with conscious perception. Would you:*

a) Report the test as $p = .03$ and conclude there is subliminal perception for this method?

b) Note that all tests are nonsignificant when a Bonferroni-corrected significance value of .05/5 is used, and conclude that subliminal perception does not exist by any of these methods?

c) Regard the strength of evidence provided by these data for subliminal perception produced by Continuous Flash Suppression to be the same regardless of whether or not four other rather different methods were tested?

orthodox statistics differ from Bayesian inference. Next, I show that Bayesian inference follows from the axioms of probability, which motivate the "likelihood principle" of inference. I explain how the orthodox answers to the scenarios in the test violate the likelihood principle and, hence, the axioms of probability. Then the contrast between Bayesian and orthodox approaches to statistics is framed in terms of different notions of rationality. Because orthodox statistics violate the likelihood principle, orthodox inference is irrational on a key intuitive notion of rationality. Finally, I explain how to conduct a Bayesian analysis, using free simple online software, to enable the most rational inferences from the data.

The Contrast: Orthodox Versus Bayesian Statistics

The orthodox logic of statistics, as developed by Jerzy Neyman and Egon Pearson in the 1930s, starts from the assumption that probabilities are long-run relative frequencies (Dienes, 2008). A long-run relative frequency requires an indefinitely large series of events that constitutes the *collective* (von Mises, 1957); the probability of some property (q) occurring is then the proportion of events in the collective with property q. For example the probability of having black hair is the proportion of people in a well-defined collective (e.g., people living in England) who have black hair. The probability applies to the whole collective, not to any one person. Any one person either has black hair or not. Further, that same person may belong to two different collectives that have different probabilities: For example, the probability of having black hair is different for Chinese people in England than for all people in England, even though a large number of people will belong to both collectives.

Long-run relative frequencies do not apply to the truth of individual theories because theories are not collectives—theories are just true or false. Thus, when using this approach to probability, the null hypothesis of no population difference between two particular conditions cannot be assigned a

probability—it is either true or false. But given both a theory and a decision procedure, one can determine a long-run relative frequency with which certain data might be obtained, which we can symbolize as *P*(data | theory and decision procedure). For example, given a null hypothesis and a procedure that includes rejection if the *t* value exceeds 2, we can work out the frequency with which we would reject the null hypothesis.

The logic of Neyman Pearson (orthodox) statistics is to adopt decision procedures with known long-term error rates (of false positives and false negatives) and then control those errors at acceptable levels. The error rate for false positives is called *alpha*, the significance level (typically.05), and the error rate for false negatives is called *beta*, where beta is 1 – power. Thus, setting significance and power controls long-run error rates. An error rate can be calculated from the tail area of test statistics (e.g., tail area of *t* distributions) adjusted for factors that affect long-run error rates, like how many other tests are being conducted. These error rates apply to decision procedures, not to individual experiments. An individual experiment is a one-time event, so it does not constitute a long-run set of events, but a decision procedure can in principle be considered to apply over an indefinite long-run number of experiments.

The probabilities of data given theory and theory given data

The probability of a theory being true given data can be symbolized as *P*(theory | data), and that is what many of us would like to know. This is the inverse of *P*(data | theory), which is what orthodox statistics tells us. One might think that if orthodox statistics indicates *P*(data | theory) it thereby directly indicates *P*(theory | data). But one cannot infer one conditional probability just by knowing its inverse. For example, the probability of being dead given that a shark has bitten one's head clean off, *P*(dead | head bitten clean off by shark), is 1. But the probability that a shark has bitten one's head clean off given that one is dead, *P*(head bitten off by shark | dead), is very close to zero. Most people die of other causes.

What applies to sharks biting heads off also applies to null hypotheses. The significance value, a form of *P*(data | theory), does not by itself indicate the probability of the null, *P*(theory | data). The particular *p* value obtained also does not indicate the probability of the null. Let us say you construct a coin heavily weighted on one side so that it will land "heads" 60% of the time. You give it to a friend for a betting game. He wishes to test the null hypothesis that it is a fair coin at the 5% significance level. He throws it five times, and gets three heads. Assuming the null hypothesis that it will land heads 50% of the time, the probability of three or more heads is 0.5. This is obviously not significant at the 5% level, even one tailed. He decides to accept the null hypothesis (as the result is nonsignificant) and also incorrectly concludes the null hypothesis has a 50% probability of being true (based on the *p* value), or a 95% probability (based on the significance level used). But you know, because of the way you constructed the coin, that the null is false, and obtaining three heads out of five throws should not change your mind about that (in fact, this nonsignificant result should give you less confidence in the null hypothesis than in your hypothesis that the coin produces heads 60% of the time). You quite rationally do not assign the null hypothesis a probability of 50% (nor 95%). When people directly infer a probability of the null hypothesis from a *p* value or significance level, they are violating the logic of Neyman Pearson statistics. Such people want to know the probability of theories and hypotheses. Neyman Pearson does not directly tell them that, as the example illustrates.

Bayesian statistics starts from the premise that we can assign degrees of plausibility to theories, and what we want our data to do is to tell us how to adjust these plausibilities. (We will discuss below why these plausibilities turn out to be probabilities—i.e., numbers obeying the axioms of probability.) When we start from this assumption, there is no longer a need for the notion of significance, *p* value, or power.[1] Instead, we simply determine the factor by which we should change the probability of different theories given the data. And arguably this is what people wanted to know in the first place. Table 1 illustrates some differences between hypothesis testing by comparing a *t* test with a Bayesian statistic called the Bayes factor, which we will describe in detail later. (Note that I will not discuss confidence intervals and credibility intervals, the Bayesian equivalent to a confidence interval, in this article; see Dienes, 2008, and Kruschke 2010b, 2011, for detailed discussion and calculations.)

The likelihood

In the Bayesian approach, probability applies to the truth of theories. Thus, we can answer questions about *p*(*H*), the probability of a hypothesis being true (our *prior probability*), and also *p*(*H* | *D*), the probability of a hypothesis given data (our *posterior probability*)—neither of which we can do when using the orthodox approach. The probability of obtaining the exact data given the hypothesis is the *likelihood*. From the axioms of probability, it follows directly that:[2]

Posterior is given by likelihood times prior.

From this theorem (Bayes' theorem) comes the *likelihood principle*: All information relevant to inference contained in data is provided by the likelihood (e.g., Birnbaum, 1962). When we are determining how given data changes the relative probability of our different theories, it is only the likelihood that connects the prior to the posterior.

The likelihood is the probability of obtaining the exact data obtained given a hypothesis, *P*(*D* | *H*). This is different from a *p* value, which is the probability of obtaining the same or more extreme data given both a hypothesis and a decision procedure. Thus, a *p* value for a *t* test is a tail area of the *t* distribution (adjusted according to the decision procedure); the corresponding likelihood is the height of the distribution (e.g., *t* distribution) at the point representing the data—not an area, and certainly not an area adjusted for the decision procedure. In

Table 1. Some Differences Between a *t* Test and a Bayes Factor

Question	Orthodox	Bayes
What are you testing?	Hypothesis vs. null hypothesis when power and confidence intervals are used appropriately; otherwise just null hypothesis.	Hypothesis vs. a contrasting hypothesis (could be null).
What summary of the data is needed to conduct the test?	*M* and *SE*.	*M* and *SE*.
What other information is needed to conduct the test?	None, the t value is just *M* / *SE*. People should determine power, and this would also require specifying the size of effect predicted by the theory, though specifying predicted effect size is not needed to get a *t* value.	A specification of the plausibility of the different effect sizes allowed by the theory.
What other criteria must be set to make a decision?	The significance level. To know the sensitivity of the test, a power should be decided, though this is not typically done.	What size Bayes factor represents strong evidence.
How are predicted effect sizes specified?	If they are specified for a *t* test, a single finite minimal expected value must be given for power to be calculated (or confidence intervals used) to assess a null result.	As a probability distribution. A single value can be specified or a range. A range can allow indefinitely small effects, which would render Neyman Pearson power calculations impossible.
What are the range of results?	A *t* value can range from 0 through 1 (consistent either with the null hypothesis or with the test being insensitive), to very large numbers, to infinity (inconsistent with the null hypothesis).	A Bayes factor varies from 0 (overwhelming support for the contrasting hypothesis) through 1 (test insensitive: equal support for both hypotheses) to infinity (overwhelming support for hypothesis).
What can you conclude?	Can assert acceptance or rejection of null hypothesis, but strangely such assertions have no implications for how confident you should be in them in any one case. (The null hypothesis can only be accepted when power or confidence intervals have been used appropriately.)	You can adjust your confidence in your hypothesis relative to the contrasting one by the amount specified by the Bayes factor.

orthodox statistics, adjustments must be made to the tail area because they accurately reflect the factors that affect long term error rates of a decision procedure. Thus, we can represent a *p* value as *P*(obtained data or data more extreme | null hypothesis and decision procedure); the likelihood for the null is *P*(obtained data | null hypothesis).

The likelihood principle may seem a truism; it seems to just follow from the axioms of probability. But in orthodox statistics, *p* values are changed according to the decision procedure: under what conditions one would stop collecting data, whether or not the test is post hoc, or how many other tests one conducted. None of these factors influence the likelihood. Thus, orthodox statistics violates the likelihood principle. I will consider each of these cases because they have been used to argue Bayesian inference must be wrong, given that we have been trained as researchers to regard these violations of the likelihood principle to be a normative part of orthodox statistical inference. But these violations of the likelihood principle also lead to bizarre paradoxes. I argue that when the full context of a problem is taken into account, the arguments against Bayes based on these points fail.

The Bayes factor

For a point of reference, a type of Bayesian analysis, the Bayes factor, is introduced, which will allow us to consider the contrast between orthodox and Bayes in detail.

The Bayes factor pits one theory against another—for example, Theory1 against Theory2. Theory1 could be your pet theory put to test in an experiment; Theory2 could be the null hypothesis or some other sort of default position. If your personal probability of Theory1 being true before the experiment is *P*(Theory1) and that for Theory2 is *P*(Theory2), then your *prior odds* in favor of Theory1 over Theory2 is *P*(Theory1)/*P*(Theory2). These prior probabilities and prior odds can be entirely personal or subjective; there is no reason why people should agree about these before data are collected if they are not part of the publically presented inferential procedure. We will present a technique here in which these priors do not figure in the inferential procedure (see also Wetzels et al., 2011). If the priors form part of the inferential procedure, they must be fairly produced and subjected to the tribunal of peer judgment (e.g., Kruschke, 2011).

Once data are collected we can calculate the likelihood for each theory. These likelihoods are things we want researchers to agree on; thus, any probabilities that contribute to them should be plausibly or simply determined by the specification of the theories. The Bayes factor (*B*) is the ratio of the likelihoods. Then, from the axioms of probability[3]

$$\text{Posterior odds} = B \times \text{prior odds.}$$

If *B* is greater than 1, then the data supported your experimental hypothesis over the null. If *B* is less than 1, then the data supported the null hypothesis over the experimental one. If *B* is

about 1, then the experiment was not sensitive. Jeffreys (1961) suggests Bayes factors above 3 or below one third are "substantial," though the evidence is continuous and there are no thresholds as such in Bayesian theory. Jeffreys suggests that for "moderate numbers of observations" (p. 435) in typical situations, his Bayesian conventions and the convention of 5% significance will often agree, meaning that his conventions provide appropriate calibration between the Bayesian and Neyman Pearson approaches, as far as such calibration can exist. Agreement between Bayes and Neyman Pearson varies according to conditions: Wetzels et al. (2011) show empirically that a special sort of Bayes factor, the Rouder–Bayes factor, will often regard evidence as slim when $p < .05$. But as we will see later in this article, when the specific theories of authors are taken into account while calculating the Bayes factor, Jeffreys's conventions match the standards of evidence that psychologists are used to accepting (see the Appendix; see also Kruschke, 2011).

Importantly, B automatically gives a notion of sensitivity; it directly distinguishes data supporting the null from data uninformative about whether the null or your theory was supported.

Table 1 shows some differences and similarities between t tests and Bayes factors. Note especially how the tests differ in terms of the range of results they produce. For both p values associated with a t test and for B, if the null is false, as number of subjects increases, then test scores are driven in one direction: p values are expected to become smaller, and both t and B values are expected to become larger. But as we will see below, when the null hypothesis is true, p values are not driven in any direction—only B is, and it is driven to zero. This distinction is crucial.

I will now consider the reasons for preferring Bayes over Neyman Pearson. I will initially examine the reasons based on the ways in which Neyman Pearson depart from the likelihood principle; then, I will consider how both Neymen Pearson and Bayes use predicted effect sizes. Neyman Pearson statistics are sensitive to factors inferentially irrelevant according to the likelihood principle: the stopping rule, whether a prediction was planned or post hoc, and how many other tests were conducted. "But surely" you might think, "inference should be sensitive to these factors?" These are the factors considered in the quiz; in answering the quiz, what were your intuitions? Did they really match what you have been taught?

Your Answers to the Quiz and Problems With the Neyman Pearson Approach

The problems with the Neyman Pearson approach, in terms of how it violates the likelihood principle, can be highlighted by considering the situations we started with (Box 1) and what your intuitions were in responding to those situations. By considering concrete situations, you may initially favor Neyman Pearson for the cases we have been trained to regard as important, namely post hoc versus planned comparisons and multiple testing. Let us see if we can dismantle those intuitions.

1. Stopping rule

For Question 1, regarding "topping up" subject numbers, I suspect a majority of researchers have at some time chosen (a) as their answer in similar cases: They have reported the topped-up data set without taking into account that the initial planned number of subjects was lower than the topped-up number. This is also the answer one might pick with a meta-analytic mind set, but use of meta-analysis here is complicated by the fact the stopping rule was conditional upon obtaining a significant finding. Answer (c) spells out the intuition motivating the choice of (a), but only Bayes provides the tools for implementing it. The Neyman Pearson tools are invalid.

On the Neyman Pearson approach one must specify the *stopping rule* in advance (i.e., the conditions under which one would stop collecting data). Once those conditions are met, there is to be no more data collection. Typically, this means one should use a power calculation to plan in advance how many subjects to run. Running subjects until a significant result is obtained is forbidden, because this will always succeed, given sufficient time, even if the null is true (e.g., Armitage, McPherson, & Rowe, 1969). Further, one cannot plan to run 30 subjects, find a p of .06, and then run 10 more, and report the p value of .04 for the full set of 40 subjects and declare it significant at the 5% level. Five percent is an inaccurate reflection of the error rate of the decision procedure because there were two chances at declaring significance. Remember that probabilities in the Neyman Pearson approach are long-run relative frequencies and thus do not apply to the individual experiment. A Type I error will be made 5% of the time at the first test; the second test can only increase the percentage of Type I errors. Each test must be conducted at a lower significance level for the overall error rate to be kept at .05 (see Armitage, Berry, & Mathews, 2002, pp. 615–623, for examples of corrections). Similarly, if you would have stopped running subjects had it been significant when you peeked at the results halfway through data collection, the actual long term error rate is not 5%, even if you end up running the exact number of participants you had originally planned!

This puts one in an impossible moral dilemma if, having tested once at the 5% level, an experiment yields a p of .06. One cannot reject the null on that number of subjects, yet one cannot accept it either (no matter what the official rules are, would you accept the null for a p of .06 when you have predicted an effect?). One cannot publish the data, yet one cannot in good heart bin the data and waste public resources. That would be immoral.

Researchers might justify "topping up" because in their hearts they believe in the likelihood principle. Surely, the subjective intentions concealed in the researcher's mind are irrelevant when drawing inferences from data—what matters is just what the obtained data are. But one cannot believe the likelihood principle and follow Neyman Pearson techniques. Long-term error rates using significance tests are affected by counterfactuals (when would you have stopped, even if you didn't?), even if likelihoods are not.[4]

The Bayes factor behaves differently from *p* values as more data are run (regardless of stopping rule). For a *p* value, if the null is true, any value in the interval 0 to 1 is equally likely no matter how much data you collect (Rouder et al., 2009). For this reason, sooner or later, you are guaranteed to get a significant result if you run subjects long enough and stop when you get the *p* value you want (e.g., Wagenmakers, 2007). When the null is true, as the number of subjects increases, the *p* value is not driven to any particular value. In contrast, as the number of subjects increases and the null is true, or closer to the truth than your alternative, the Bayes factor is driven toward zero. Savage (1962, pp. 72–73) considers a particular case in which one continues to sample until a Bayes factor of 10 is achieved, despite the fact that the null is true, and shows "it is quite probable that [one] will never succeed until the end of time." That is, as in Savage's example, you could run an infinite number of subjects and, on many such experiments (at least 9 out of 10), never have *B* reach 10 (though, of course, false alarms will be obtained on 1 out of 10 of such experiments—one can never guarantee their elimination; see Dienes, 2008; Kruschke, 2011; Royall, 1997). Savage comments, "it is impossible to be sure of sampling until the data justified an unjustifiable conclusion, just as surely as it is impossible to build a perpetual motion machine. After all, whatever we may disagree about we are surely agreed that Bayes theorem is true where it applies." Hence, you can run as many subjects as you like when using Bayes and stop when you like, solving the moral dilemma just mentioned. And if this sounds more sensible, it is because it is literally more rational, as argued below.

2. Planned versus post hoc comparisons

For Question 2 (finding a good theory for predicting a set of results already obtained), I suspect many people have decided (a) in similar circumstances. They have treated the results as predicted, because of the Bayesian intuitions in (c) and so used the wrong tools for the right reasons. The introductions of many papers appear to be written entirely in the light of the results. We implicitly accept this as good practice, and indeed, we train our students to do likewise for the sake of the poor reader of the paper. But (b) is the correct answer based on the Neyman Pearson approach, and maybe your conscience told you so. But should you be worrying about what is murky (which really came first, data or hypothesis?) or, rather, about what really matters—whether the predictions really follow from a substantial theory in a clear simple way?

When using Neyman Pearson, it matters whether you formulated your hypothesis before or after looking at the data (post hoc vs. planned comparisons): Predictions made before rather than after looking at the data are treated differently. In Bayesian inference, the evidence for a theory is just as strong regardless of its timing relative to the data. This is because the likelihood is unaffected by the time the data were collected.

The likelihood principle contradicts not only Neyman Pearson on this point, but also the advice of Popper (1963) and Lakatos (1978), who valued the novelty of predictions (though Lakatos later gave up the importance of temporal novelty, Lakatos & Feyerabend, 1999, pp. 109–112). Kerr (1998) also criticized the practice of HARKing: hypothesising after the results are known. Indeed, novel predictions are often impressive as support for a theory. But this may be because the choice of auxiliary hypotheses (i.e., those hypotheses implicitly or explicitly used in connecting theory to specific predictions) was fair and simple when making novel predictions. Post hoc fitting can involve preference (for no good reason) for one auxiliary over many others of at least equal plausibility. Thus, a careful consideration of the reason for postulating different auxiliaries should render novelty irrelevant as a factor determining the evidential value of data. That is, the issue is not the timing of the data per se, but the priori probability of the hypotheses involved. And prior probability is something Bayes is uniquely well equipped to deal with.

Consider an example that has been used as a counter argument to the likelihood principle. The example seems to show that we should take into account whether a result was predicted in advance or not, which is contrary to the likelihood principle. Having considered the intuition that planned versus post hoc comparisons are inferentially important, I will show why it is false and the likelihood principle is in fact correct.

I have a pack of cards face down. I lift up the top card. It is a six of hearts. Call the hypothesis that the pack is a standard pack of playing cards *Hs*. Call the hypothesis that every card in the pack is a six of hearts *H6h*. In a standard pack, the likelihood of drawing a six of hearts is 1 out of 52. So $L(Hs) = 1/52$. If the pack consists of 52 sixes of hearts, the likelihood of drawing a six of hearts is 1. So $L(H6h) = 1$. So the Bayes factor in favor of the pack consisting only of sixes of hearts versus being a standard pack is 52. That is, the data greatly favor the former hypothesis over the latter, and this conclusion seems unreasonable at first. If you walked in a room and saw a pack of cards face down, your initial prediction would be that they are a standard pack of playing cards. The drawing of a single six of hearts would not change your mind. So the hypothesis that they are all sixes of hearts is purely post hoc, a mindless fitting of the data. If Bayesian statistics support the post hoc theory over the theory that it is a standard pack of cards, then something is wrong with Bayes. If someone could predict in advance that the pack was all sixes of hearts that would be one thing; but the point is that they would not. By missing out on the importance of what can be predicted in advance, the likelihood principle appears to fail scientists.

Remember that the likelihood principle follows from the axioms of probability. The axioms of probability are, by their nature, almost self-evident assumptions. They will not lead to wrong conclusions. Indeed, in this case, if we put the problem in its full context, we see the Bayesian answers are very sensible (see Royall, 1997, p. 13, for the following argument). Consider a situation in which, before we pick up the card, there are 52 hypotheses that the pack of cards is all of one sort of card: all aces of hearts, all twos of hearts, and so on. Call the probability that one or other of these hypotheses is true *P*. So the probability of any one of them being true is $P/52$, assuming we hold

them to all have equal probability. Once we have observed the six of hearts, all these hypotheses go to zero, except for *H6h*. The probability of that hypothesis goes to *P*. The probability that the whole pack is all of one suit remains the same—it is still *P*. The probability that it is a standard pack of cards remains the same. The axioms of probability and their consequence, Bayes theorem, give us just the right answer. There is no need to introduce an extra concern with ability to predict in advance; that concern is already implicitly covered in the Bayesian approach. It is not the ability to predict in advance per se that is important; that ability is just an (imperfect) indicator of the prior probability of relevant hypotheses. When performing Bayesian inference, there is no need to adjust for the timing of predictions per se. Indeed, it would be paradoxical to do so: Adjusting conclusions according to when the hypothesis was thought of would introduce irrelevancies into inference, leading to one conclusion on Tuesday and another on Wednesday for the same data and hypotheses.

3. Multiple testing

For Question 3, concerning whether one would modify the conclusions for one test of subliminal perception based on the fact that other methods were tested, practice may vary depending on how the author feels about subliminal perception. After all, there is no strict standard about what counts as a "family" for the sake of multiple testing (Baguley, in press). There is a pull between accepting that surely there is evidence for this method, as stated in (c), and the realization that more tests means more opportunities for inferential mistakes. But one should not confuse strength of evidence with the probability of obtaining it (Royall, 1997). Evidence is evidence, even if, as one increases the circle of what tests are in the "family," the probability that some of the evidence will be misleading increases.

When using Neyman Pearson, one must correct for how many tests are conducted in a family of tests. For example, if 100 correlations were run to test a theory and only four were just significant at the 5% level, researchers would not try to interpret those significant results. By contrast, when using Bayes, it does not matter how many other statistical hypotheses are investigated. All that matters is the data relevant to each hypothesis under investigation.

Consider an example from Dienes (2008) to first pump your intuitions along Neyman Pearson lines (to confirm the intuition that one should correct for multiple testing); then, as above, I will show how the axioms of probability do indeed give us the sensible answer and, hopefully, your intuitions come to side with Bayes (that one should not, after all, correct for multiple testing in evaluating the strength of evidence of data for a theory).

The example is about searching for the reincarnation of a recently departed lama by a search committee set up by the Tibetan Government-in-exile. The lama's walking stick is put together with a collection of 20 others. Piloting at a local school shows each stick is picked equally often by children in general. We have now set up a test with a known and acceptable testwise Type I error probability, controlled to be less than 5% for each individual test. If a given candidate picks the stick, $p = 1 / 21 < .05$. Various omens narrow the search down to 21 candidate children. They are all tested and one of these passes the test. Can the monks conclude that he is the reincarnation?

The Neyman Pearson aficionado says, "No. With 21 tests, the family-wise error rate is $1 – (20/21)^{21} = 0.64$. This is unacceptably high. Of course, if you test enough children, sooner or later one of them will pass the test. That proves nothing. As Bayes does not correct for multiple testing, surely the Bayesian approach must be wrong."

The Bayesian responds, "Assume the reincarnation will definitely choose the stick. If the 10th child chose the stick, the Bayes factor, *B*, for the 10th child = 21. Whatever your prior odds that the 10th child was the reincarnation, they should be increased by a factor of 21."

The Neyman Pearson aficionado feels his point has been made. "You have manufactured evidence out of thin air! By ignoring the issue of multiple testing, you have found strong evidence in favor of a child being the reincarnation just because you tested many children" (see e.g., Mayo, 1996, 2004, for the need for correction for multiple testing as an argument against Bayes). Hopefully you now strongly feel that one should correct for multiple testing.

The Bayesian patiently continues with an argument similar to the one in the previous section, "The likelihood of any child who did not choose the stick is 0. Call the prior probability that one or other of these children was the reincarnation *P*. If prior probabilities for each individual child are equal, the prior probability that any one is the reincarnation $= P / 21$. After data, 20 of these go to zero. One goes to $21 \times P / 21 = P$. The posterior probabilities still sum to *P*. If you were convinced before collecting the data that the null was false you can pick the reincarnation with confidence; conversely, if you were highly confident in the null beforehand you should be every bit as confident afterward. And this is just as it should be!"

The Bayesian answer does not need to correct for multiple testing because an answer does not need to be corrected if it is already right. Once one takes into account the full context, the axioms of probability lead to sensible answers, just as one would expect. As I point out in Dienes (2008), a family of 20 tests in which one is significant at the .05 level typically leads one by Bayesian reasoning to have more confidence in the family-wise null hypothesis that "all nulls are true" while decreasing one's confidence in the one null that was significant.[5] And this fits one's intuitions that if evidence went against the null in only 4 out of 100 correlations, one would be more likely to think the complete null is true, but still find oneself more likely to reject the null for the four specific cases. If all 100 correlations bore on a theory that predicted nonzero correlations in all cases, then one's confidence in that theory would typically decrease by a Bayesian analysis.

The moral is that in assessing the evidence for or against a theory, one should take into account all the evidence relevant to the theory and not cherry pick the cases that seem to support it. Cherry picking is wrong on all statistical approaches. A large

Table 2. Contrasts Between Bayesian and Orthodox Statistics Following From Whether the Likelihood Principle Is Obeyed

Factor	When you initially intended to stop running participants	Whether or not you predicted a result in advance of obtaining it	The number of tests that are grouped in a family
Orthodox: Could this factor affect whether or not a null hypothesis is rejected?	Yes	Yes	Yes
Bayes: Does this factor ever affect the support of data for a hypothesis?	No. You can always run more participants to acquire clearer evidence if you wish	No. No one needs to second guess which really came first	No. Test as many different hypotheses as is worth your time, but take into account all evidence relevant to a theory

Note: Because orthodox statistics are sensitive to the factors listed, contrary to the likelihood principle, different people with the same data and hypothesis may come to opposite conclusions in each case.

number of results showing evidence for the null against a theory still count as against the theory, even if a few of the effects the theory predicted are supported. And Bayes gives one the apparatus for combining such evidence to come to an overall conclusion—an apparatus missing in Neyman Pearson. Thus, it is the Bayesian approach, rather than the Neyman Pearson approach, that is most likely to demand that researchers draw appropriate conclusions from a body of relevant data involving multiple testing. Bayes factors close to zero count as evidence against the theory; in practice, using orthodox statistics, nonsignificant values are either used as evidence against the theory or not depending on whim.

Key differences between the approaches that follow from the likelihood principle are shown in Table 2. To quickly summarize the argument in Table 2, the orthodox approach is irrational because different people with the same data and same hypotheses could come to different conclusions.

The Rationality of the Bayesian Approach

What is it to be rational? One definition of rationality is having sufficient justification for one's beliefs, and another is that it is a matter of having subjected one's beliefs to critical scrutiny. Popper and others inspired by him followed the latter definition and termed it *critical rationalism* (e.g., Miller, 1994; Popper, 1963). In this view, there is never a sufficient justification for a given belief because knowledge has no absolute foundation. Propositions can be provisionally accepted as having survived criticism, given other propositions those people in the debate are conventionally and provisionally willing to accept. All we can do is set up (provisional) conventions for accepting or rejecting propositions. An intuition behind this approach is that irrational beliefs are just those not subjected to sufficient criticism (consider any of your colleagues' irrational beliefs!).

Critical rationalism bears some striking similarities to the orthodox approach to statistical inference—the Neyman Pearson approach. In this view, statistical inference cannot tell you how confident to be in different hypotheses; it only gives conventions for behavioral acceptance or rejection of different hypotheses, which, given a relevant statistical model (which can itself be subjected to testing), results in controlled, preset long-term error rates. One cannot say how justified a particular decision is or how probable a hypothesis is, and one cannot give a number to how much data supports a given hypothesis (how justified the hypothesis is, or how much its justification has changed)—one can only say that the decision was made by a decision procedure that in the long-run controls error frequencies.

Now consider the first definition of rationality: having sufficient justification for one's beliefs. If we want to assign continuous degrees of justification (i.e., belief) to propositions, what are the rules for logical and consistent reasoning? Cox (1946; see Halpern, 1999; Jaynes, 2003, chapter 2; Sivia & Skilling, 2006, for a full discussion) chose two minimal desiderata, namely that

1. If we specify degree of belief in *P*, we have implicitly specified degree of belief in not-*P*,
2. If we specify degree of belief in *P* and also specify degree of belief in *Q* given *P*, then we have implicitly specified degree of belief in (*P* and *Q*).

Cox did not assume in advance what form this specification was nor what the relationships were—just that the relationships existed. Using deductive logic, Cox showed that degrees of belief must follow the axioms of probability if we wish to accept the above minimal constraints. Thus, if we want to determine by how much we should revise continuous degrees of belief, we need to make sure our system of inference obeys the axioms of probability.[6] If researchers want to think in terms of the degree of support data provide for a hypothesis, they should make sure their inferences obey the axioms of probability.

One version of degrees of belief are *subjective probabilities*, personal convictions in an opinion (e.g., Howson & Urbach, 2006). When probabilities of different propositions form part of the inferential procedure we use in deriving conclusions from data, then we need to make sure that the procedure is fair. Thus, there has been an attempt to specify *objective probabilities* that follow from the informational specification of a problem (e.g., Jaynes, 2003). In this way, the probabilities become an objective part of the problem, with values that can be argued about, given the explicit assumptions, and that do not depend any further on personal idiosyncrasies. Thus, these sort of

probabilities can be regarded as consistent with critical rationalism (despite Popper's aversion to Bayes).

In sum, one notion of rationality is having sufficient justification for one's beliefs. If one can assign numerical continuous degrees of justification to beliefs, then some simple minimal desiderata lead to the "likelihood principle" of inference. Hypothesis testing violates the likelihood principle, indicating that some of the deepest held intuitions we train ourselves to have as orthodox users of statistics are irrational on a key intuitive notion of rationality. For those who want to be rational in the sense of giving a degree of justification to their conclusions, the details of Bayes factors in practice are now considered.

Effect Size

Thus far, I have focused on how orthodox statistical inference is influenced by factors that are irrelevant for inference (according to the likelihood principle); next, we consider the converse problem—namely, how typical use of statistics is often not influenced by a factor that is logically relevant to inference: effect size. We need to consider the issue of effect size in order to discuss how to calculate a Bayes factor in practice. Bayes factors demand consideration of relevant effect sizes.

A problem in many areas is that researchers have been relating theories to statistics by using the wrong question: "Is there a difference?" (or "Is there an association?"), with acceptable answers being "yes," "no," or "withhold judgment," depending on data and statistical persuasion. The question has the virtue of simplicity. Fisher argued that the only possible answers were "yes" (after a significant result, one can conclude there is a difference) or "withhold judgment" (after a nonsignificant result; see Baguley, in press; Dienes, 2008; Wright, 2010, for overviews of Fisherian and Neyman Pearson inference). A nonsignificant result does not allow a definitive conclusion, because there might be a population difference that the test was not sensitive enough to pick up.

Neyman developed two specific measures of sensitivity: power and confidence intervals. A confidence interval is the set of population values that the data are consistent with. For any continuous measure based on a finite number of subjects, an interval cannot be an infinitesimally small point: It may include zero but must include other values too. So a null result is always consistent with population values other than zero—indeed, it is always consistent with population values on either side of zero. That is why a nonsignificant result cannot on its own lead to the conclusion that the null hypothesis is true; that's why, following Fisher, one can never accept the null hypothesis: One can only answer "yes" or "withhold judgment."

However, theories and practical questions generally specify, even if vaguely, relevant effect sizes. And they must, if predictions of a difference are ever to be tested (as in potentially falsified). The research context, if expertly known, usually provides a range of effects that are too small to be relevant and a range of effects that are consistent with theory or practical use. The real research question is not the simple question with which I started this section, but rather "Can we distinguish the range of effects predicted or required by the main research problem, on the one hand, and those that are too small or otherwise inconsistent, on the other?"

Researchers may initially believe they do not know what those ranges or predictions are because they are not used to thinking that way. But, arguably, researchers do have relevant intuitions, and that is why it has made sense to them to assert null hypotheses. If a researcher says "Look, those means really are very close together," then they have an implicit scale of relevant effect size. It is just that they have not made all their assumptions explicit. It is time we related our theories to statistics via questions that allow the answers we need and time we made all relevant assumptions explicit. If we want to use null results in any way to count against theories that predict an effect, we (logically) must consider the range of effect sizes consistent with the theory.

Effect size is very important in the Neyman Pearson approach: One must specify the sort of effect one predicts in order to calculate power (this is why one can assert the null hypothesis on the Neyman Pearson approach).[7] On the other hand, Fisherian significance testing leads people to ignore effect sizes. People have followed Fisher's methods, while paying lip service to effect sizes, but not heeding Fisher's advice that nothing follows from a null result. By contrast, one must specify what sort of effect sizes a theory predicts to calculate a Bayes factor. Because it takes into account effect size, the Bayes factor distinguishes evidence that there is no relevant effect from no evidence of a relevant effect. One can only confirm a null hypothesis when one has specified the effect size expected on the theory being tested.

In specifying theoretically expected effect sizes, we should ask ourselves "What size effect does the literature suggest is interesting for this particular domain?" Rather than following the common practice of plucking a standardized effect size of 0.5 out of thin air, researchers should get to know the data of the field. Sometimes, when one really does not know what sort of effect to expect, using wild speculations—like a Cohen's d of 0.5 because that is the sort of effect psychologists in general often deal with—may be the best one can do. But most of the time, researchers can do better. (For arguments for the frequent relevance of raw rather than standardized effect sizes, see Baguley, 2009; Ziliak & McCloskey, 2008.)

Despite some attempts to encourage researchers to use confidence intervals, their use has not taken off (Coulson, Healey, Fidler, & Cumming, 2010; Fidler, Thomason, Cumming, Finch, & Leeman, 2004; Oakes, 1986). Confidence intervals of some sort would deal with many problems (either confidence, credibility, or likelihood intervals; see Dienes, 2008, for definitions and comparison). But an approach that has a flavor of a t or other inferential test might be accepted more readily. Further, confidence intervals themselves have all the problems enumerated above for Neyman Pearson inference in general (unlike credibility or likelihood intervals): Because confidence intervals consist of all values nonsignificantly different from the sample mean, they inherit the arbitrariness of significance

testing (e.g., Kruschke, 2010a). So I urge the use of the Bayes factor for key tests in the context of theory testing or determining practical significance of an effect.[8]

How to Calculate a Bayes Factor

To calculate a Bayes factor in support of a theory (relative to the null hypothesis, for example), one has to specify what the probability of different effect sizes are, given the theory. In a sense, this is not new: Researchers should specify predicted effect sizes in any case. And Bayes gives us the apparatus to flexibly deal with different degrees of uncertainty regarding the predicted effect size. Logically, one needs to know what a theory predicts in order to know how much it is supported by evidence.

Dienes (2008) provides a Bayes factor calculator, which can be found as a Flash program on the book's website. The book and website also provide the MatLab code for the Bayes factor, and Baguley and Kaye (2010) provide corresponding *R* code. The calculator requires two things: First, a summary of the data and, second, the predictions of the theory.

What you need to enter into the Bayes factor calculator

In terms of the data, the Bayes factor calculator asks for a mean together with its standard error. The program assumes that the sampling distribution of the mean is roughly normal. For example, for the equivalent of a *t* test, the mean entered would be the mean difference between conditions and the standard error would be the standard error of this difference. If you know the *t* for the data, the relevant standard error can be easily obtained as (mean difference) / *t*, whether the *t* is repeated measures, between subjects, or one sample.

In terms of predictions of the theory (or requirements of a practical effect), one has to decide what range of effects are relevant to the theory (or practical situation). The program allows one to specify the range in three ways. First, one could provide a uniform distribution—that is, all values between a lower bound and an upper bound. All values within the bounds are represented as possible and equally likely given the theory and all those outside are inconsistent with the theory. Second, one could provide a normal distribution, in which one value is the most likely given the theory (e.g., a value often found in this context), and any values lower or higher are progressively less likely. One needs to specify the mean and standard deviation of this two-tailed normal distribution. Third, one could specify a normal distribution centered on zero with only one tail. The theory predicts an effect in one direction, but smaller values are generally more likely than larger values (e.g., consider a telepathy experiment). One needs to specify the standard deviation of this one-tailed normal distribution. See Dienes (2008) and that book's website for more explanation. Hopefully, these three distributions capture the predictions of most theories. The hard part is determining the best way to represent the predictions of a theory: which of these distributions and with what parameters? But it is precisely the careful consideration of this question for each research domain—done with thought and not with an automatic default—that will mean we are connecting theory to data with the right statistical question. (The Appendix provides some rules of thumb.)

Examples of the three distributions

Consider a theory that predicts that a difference will be in one direction. A minimally informative distribution, containing only the information that the difference is positive, is to say all positive differences are equally likely between zero and the maximum difference allowed by the scale used. Such a vague prediction works against finding evidence in favor of the theory. Generally, researchers can do better than that. For example, consider a learning situation in which there is a standard set of materials and test performance is generally about 65% (where chance is 50%)—for example, implicit learning of a certain artificial grammar that has been extensively investigated before. People are exposed to strings of letters that follow a set of rules but are not told of the rules. Then, they are told of the existence of the rules and classify new strings in a test phase. In our new experiment, we speculate that asking people to work out the rules of the grammar for 5 min after the training, but before the test, should reduce performance: We speculate that the conscious thinking will interfere with the unconscious knowledge. Because performance on average will be about 65% without this intervening task, the maximum reduction is 15%. Thus, we might use a uniform distribution for the size of reduction with equal probability between 0% and 15% reduction (rather than the implausible 0%–50%). If the actual mean reduction is 5%, with a standard error of 2.5%, *t* would be equal to 2 (just about significant, depending on degrees of freedom). Entering these four numbers ($M = 5\%$ and $SE = 2.5\%$; upper and lower bounds of mean difference predicted by theory = 0% and 15%) into the online calculator reveals that the Bayes factor in favor of the theory (that a reduction will occur) over the null hypothesis is 3.02, substantial evidence for the theory over the null hypothesis (by Jeffreys's, 1961, conventions, i.e., Bayes factor greater than 3).

We may be able to make the predictions more precise by relating the manipulation to a previous one and suggesting the same mechanisms are at work. For example, previous work may have shown that being informed of the existence of rules and trying to work them out during the training phase reduces performance by 5%. By speculating that the same mechanism is at play for the current manipulation (thinking about the rules after training rather than during), one could argue that a reduction in performance of 5% would be most likely but by no means certain. In fact, we might think any decrement from 0% to 10% is reasonably plausible. The probability density must be close to zero for a reduction in performance of 0%, so we could define the predictions by a normal distribution with a mean of 5% and a standard deviation of 2.5%. Notice that the most likely predicted effect according to the theory, based on identifying the mechanism of change as the same as in some

relevant past research, is as found (5%). We benefit from this and obtain a Bayes factor of 5.22. Both analyses qualitatively agree, but the more precisely we can predict an effect by bringing to bear past research and identifying mechanisms, the stronger the data can support the hypothesis (and conversely the easier it would be to falsify the theory).

Finally, one could argue that smaller effects are more likely than larger ones. This can be modeled by one half of a normal distribution, with its mode at zero and its tail dropping away in the positive direction. As similar sorts of effects as those predicted in the past have been on the order of a 5% difference between conditions in classification accuracy (as in our example), then the standard deviation of the normal distribution can be set to 5%. This distribution would imply that smaller effects are more likely than bigger ones and that effects bigger than about 10% are unlikely. With these assumptions, the Bayes factor is 4.27. Once again, a very similar conclusion is reached, giving confidence in the qualitative conclusion: The evidence supports the theory of interference over the null hypothesis of no difference.

Remember that by specifying these distributions (uniform, normal, half-normal) you are not saying what the distribution of the data should look like: You are specifying the plausibility of the population mean given a theory.[9]

A Bayes factor compares one theory with another; thus, several Bayes factors could be calculated to determine the relative support for different theories. For example, if we take the theory that there is some change in performance, but an increase is as likely as a decrease, we could represent predictions by a normal two-tailed distribution centered on zero with a standard deviation of 5. Because this theory allows more than the one-tailed theory, it is only barely supported by the data: The Bayes factor is 2.22, which is not substantial evidence for the theory over the null. In general, theories that predict directions of effect corresponding to those found in single experiments will fare better than theories that do not, just as they should.

Bayes factors vary according to assumptions, but they cannot be made to vary ad lib: Often a wide range of assumptions for spelling out a particular theoretical intuition leads to essentially the same conclusion (consider the first three Bayes factors above). Importantly, the assumptions are open to public scrutiny. They can be debated and other assumptions used according to the debate. In this sense, Bayes is objective. In Neyman Pearson inference, the inference depends on how the experimenter decided to stop, when he or she thought of the hypothesis, and what other hypotheses the researcher or the research assistant might have tested. These concerns are not open to public scrutiny and may not even be known by the experimenter.

Examples of how Bayes factors relate to significance tests

Because Bayes factors connect theory to data in appropriate ways (i.e., by taking into account effect size), Bayes puts the results of significance testing into perspective. Consider a theory that predicts a difference between conditions. A Bayes factor can indicate there is more support for the null hypothesis than for the theory after a significant result. And, vice versa, a Bayes factor can indicate more support for the theory than for the null hypothesis after a nonsignificant result (even if power is controlled). Because the distribution of effects predicted by a theory depends on the theory, no firm rules can be given for when orthodox and Bayesian answers will differ in this respect. It all depends on the theory considered (cf. Berger, 2003).

Consider the theory that prejudice between ethnic groups can be reduced by making both racial groups part of the same ingroup. A manipulation for reducing prejudice following this idea could consist of imagining being members of the same sports team. A control group could consist of imagining playing a sport with no mention of the ethnic group. A postmanipulation mean difference in prejudice (in the right direction) of x raw units is obtained with 30 participants, equal to the standard error of difference; a nonsignificant t value of 1.00 is obtained. What follows from this null result? Should one reduce one's confidence in the theory (assuming the experiment is regarded as well designed)? It depends. Let us say that instead of imagining the scenario, participants actually engaged in a common activity and that a $2x$ reduction in prejudice on the same scale was obtained. It seems unlikely that imagination would reduce prejudice by more than the real thing. If smaller effects are regarded as more likely than larger effects in general, then we may model predictions by half a normal, with its mode on zero, and a standard deviation of x units. In this case, the Bayes factor is 1.38. The data are essentially uninformative, but if anything we should be more confident in the theory after getting these null results. It would be a tragic mistake to reject the usefulness of imagination treatments for prejudice based on this experiment. Indeed, even if the mean difference had been exactly zero, the Bayes factor is 0.71, and one's confidence in the theory relative to the null should be barely altered. Even if the mean difference had been x in the wrong direction, the Bayes factor is still 0.43.[10] This does not count as substantial evidence against a theory by Jeffreys's (1961) suggested convention for Bayes factors of more than 3 (or less than a 1/3) for indicating substantial evidence. Indeed, if one felt strongly confident of the theory before collecting the data, one could normatively still be very confident afterward. In sum, Bayes factors, but not orthodox statistics, tell us when there is no evidence for a relevant effect and when there is evidence against there being a relevant effect.

Different ways of using Bayes factors

For a couple of other ways of using Bayes factors, see Rouder et al. (2009) and Wetzels et al. (2011) for a suggested "default" Bayes factor to be used on any data where the null hypothesis is compared with a default theory (namely, the theory that effects may occur in either direction, scaled to a large standardized effect size); and Kruschke (2010a, 2010b, 2011) for Bayes factors for a set of default hypotheses (much like the default effects in analyses of variance) where inference is based on the

posterior and thus takes into account the priors of hypotheses. The procedure I have been illustrating, by contrast, involves calculating Bayes factors for specific hypotheses that interest the researcher and allows priors to remain personal and not part of public inference. By following Bayes rule, each of these approaches means rational answers are provided for the given assumptions, and researchers may choose each according to their goals and which assumptions seem relevant to them.

As mentioned earlier, Bayes factors are just one form of Bayesian inference—namely, a method for evaluating one theory against another. A researcher will often also be interested in estimating a range of possible population values of the raw effect size. For this, credibility or likelihood intervals can be used (see Dienes, 2008; Kruschke, 2010b, 2011; Royall, 1997). Indeed, a researcher may use such intervals without Bayes factors if no strong theory is at stake for the test in question, or indeed if the intervals suffice in evaluating a theory. Bayes offers a range of tools for the researcher. For examples of how to write up Bayes factors for articles, see the website for Dienes (2008).

Multiple Testing and Cheating

With Bayes factors, one does not have to worry about corrections for multiple testing, stopping rules, or planned versus post hoc comparisons. But, you might insist, all these rules in orthodox statistics were there to stop cheating. If one conducts enough different tests, one can get good results just by doing enough tests. For example, different assumptions concerning the predictions of a theory lead to different Bayes factors. What is to stop a researcher picking the best one?

This concern might be partly based on a lingering feeling that there should be one legitimate Bayes factor for a set of data just as the applied user of orthodox statistics is sometimes taught that there is one right test for a given situation. However, a Bayes factor just tells you how much support given data provides for one theory over another. There is no one right Bayes factor. For example, if one theory predicts an effect in one direction and a competing theory predicts an effect in the other direction, we could represent the predictions of the first theory with a half normal distribution extending in the positive direction and the predictions of the second theory with a half normal distribution extending in the negative direction. The Bayes factor calculator I provide on my website (Dienes, 2008) will give Bayes factors for each of those specifications as compared to the null, and the ratio of the provided Bayes factors will be the Bayes factor for one theory over another. Each of these Bayes factors is informative in its own right. Naturally, there can be as many Bayes factors as comparisons between theories one wishes to make.

But what about, for example, how we choose the Bayes factor for any one theory over the null—do we use a half normal or a uniform? How do we scale each of these? With different assumptions and different scalings, we get different answers. What would prevent a researcher from cheating and just choosing the best one?

Strictly, each Bayes factor is a completely accurate indication of the support for the data of one theory over another, where the theories are defined by the precise predictions they make, as we have represented them. The crucial question is which of these representations best matches the theory as the researcher has described it and related it to the existing literature. One constraint on the researcher will be the demand for consistency: Arguing for one application of a theory ties one's hands when it comes to another application. And, of course, there is the tribunal of colleagues to get through in arguing what the theory actually predicts. One solution is to use a default Bayes factor for all occasions (Rouder et al., 2009; Wetzels et al., 2011), though this amounts to evaluating a default theory for all occasions, regardless of one's actual theory. So a default Bayes factor will only test your theory if it happens to correspond to the default. Another solution is to define the predictions according to simple procedures (see the Appendix) to ensure the theory proposed is tested according to fair criteria.

Note that there is not anything wrong with finding out which ways of representing predictions produce especially high Bayes factors. This is not cheating but determining possible constraints on theory. An orthodox statistician might feel pangs of guilt in finding out too much from the data. But absurd claims like "do not run too many subjects otherwise you might get a significant result for an effect size too small to be interesting" have no place in Bayesian statistics. In Bayes, there is no need to flagellate yourself for finding out more. The apple of knowledge tastes good. What is needed to evaluate one theory against another is (all) the relevant data and the two theories; whatever other data and other theories are evaluated is simply additional knowledge.

Finally, when using Bayes in multiple testing, one can use the fact that one is testing multiple hypotheses to inform the results if one believes that testing these multiple hypotheses is relevant to the probability of any of them being true (Westfall, Johnson, & Utts, 1997). One can use the fact that many of the tests revealed negligible effect sizes to inform other tests if one believes the effect sizes of the hypotheses are drawn from a common distribution (Kruschke, 2010b, 2011). In these cases, the other tests influence not the Bayes factor, but the prior probabilities assigned to the different hypotheses tested by each Bayes factor.

Weaknesses of the Bayesian Approach

The strengths of Bayesian analyses are also their weaknesses: First, Bayesian analyses force people to consider what a theory actually predicts, *but* specifying the predictions in detail may be contentious. Second, Bayesian analyses escape the paradoxes of violating the likelihood principle, described above, *but* in so doing they no longer control Type I and Type II errors. We discuss these points in turn.

Calculating a Bayes factor depends on answering the following question about which there may be disagreement: What way of assigning probability distributions of effect sizes as predicted by theories would be accepted by protagonists on all

sides of a debate? Answering this question might take some arguing, and this may be regarded a weakness. But isn't this just the sort of argument that psychology has been missing out on and could really benefit from (cf. Meehl, 1967)? People would really have to get to know their data and their theories better to argue what range of effect sizes their theory predicts. This will take effort compared with simply calculating *p* values. But this effort will involve thinking carefully about what specific contrasts (probably one degree of freedom) actually address the key theoretical questions of the research, and people will not churn out all the effects of an ANOVA, for example, just because they can. People will think more carefully about theoretical mechanisms so as to link experiments to past research to predict relevant effect sizes. Results sections will become focused, concise, and more persuasive.

To begin with, psychologists may start using Bayes factors only to support key conclusions, especially based on null results, in papers otherwise based on extensive orthodox statistics. Of course, it would have to be done that way initially because editors and reviewers expect orthodox statistics. And it would be good to explore the use of Bayes factors gradually in any case. Once Bayes factors become part of the familiar tool box of researchers, their proper use can be considered in the light of that experience.

An alternative response to the problem of assigning a probability distribution to effect sizes is to not take on the full Bayesian apparatus: One can just report likelihoods for the simple hypotheses that the population value is 1, 1.1, ...etc. This is "theory free" in the sense that no prior probabilities are needed for these different hypotheses (see Blume, in press; Dienes, 2008, chapter 5; Johansson, in press; Royall, 1997). This procedure results in a likelihood interval, similar to confidence interval (though one that follows the likelihood principle). The "likelihood approach" has the advantage of not committing to an objective or subjective notion of probability, and not worrying about precisely how to specify probability distributions over effect sizes, while committing to the likelihood principle. On the other hand, if a probability distribution over effect sizes can be agreed on, the full use of Bayes can be obtained (Jaynes, 2003). In particular, one can average out nuisance parameters, and assign relative degrees of support to different theories each consistent with a range of effect sizes.

The second potential weakness is that Bayesian procedures, because they are not concerned with long term frequencies, are not guaranteed to control Type I and Type II error probabilities of decision procedures (Mayo, 1996).

Royall (1997) showed how the probability of making certain errors with a likelihood ratio—or Bayes factor—can be calculated in advance. In particular, for a planned number of subjects, one can determine the probability that the evidence will be weak (Bayes factor close to 1) or misleading (Bayes factor in wrong direction). These error probabilities have interesting properties compared to Type I and II error rates. No matter how many subjects one runs, the Type I error is always the same: typically 5%. But for Bayes factors, the more subjects one runs, the smaller the probability of weak or misleading evidence. Further, these probabilities decrease as one runs more subjects, no matter what one's stopping rule is. One can always decide to run more subjects to firm up the evidence.

Nonetheless, Mayo (1996) argued that Type I and II errors are things scientists want to avoid; but a Bayesian analysis does not control them. If we want to control them, we need to use Neyman Pearson statistics (or a close variant). And then we are compelled to violate the likelihood principle.

Ultimately, the issue is about what is more important to us: using a procedure with known long term error rates or knowing the degree of support for our theory (the amount by which we should change our conviction in a theory). If we want to know the degree of evidence or support for our theory, then our reliance on orthodox statistics is irrational.

Conclusion

I suggest that the arguments for Bayes are sufficiently compelling that psychologists should be aware of the debates at the logical foundations of their statistics and make an informed choice between approaches for particular research questions. The choice is not just academic—it would profoundly affect what we actually do as researchers.

Appendix

What Does My Theory Predict?

Here are some rules of thumb for deciding how to represent what a theory predicts, but bear in mind that representing the predictions of a theory cannot be automated because for a given theory there is no telling what consideration may be relevant. These suggestions are thus not exhaustive.

1. Some scales have determinable limits (either logically determined or by background knowledge).[11] For example, if a 0 to 5 rating scale is used, the difference between conditions cannot exceed 5. Thus, if all one is willing to say is that the theory predicts that "there will be a positive difference," one could use a uniform over the range seen in Figure A1.
2. In such cases, one may feel it absurd to think that all such differences are equally plausible (as a uniform

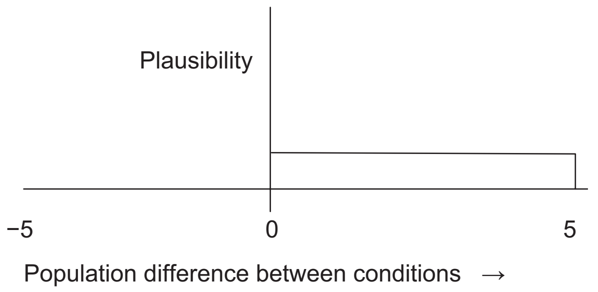

Fig. A1.

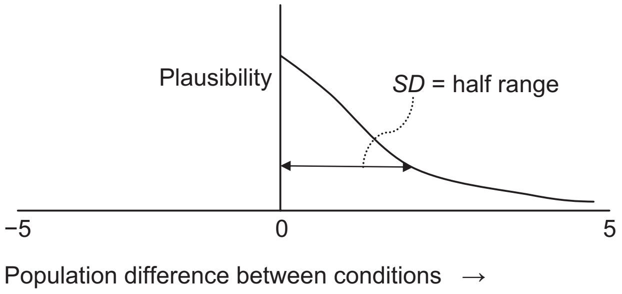

Fig. A2.

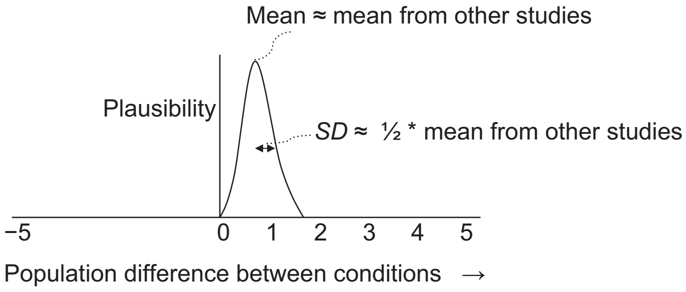

Fig. A3.

represents it); in fact, the smaller values are more likely than larger values. Then, use a half-normal distribution with a mode of zero and a standard deviation of half the range (e.g., in the example above, let $SD = 2.5$). See Figure A2.

3. Often there is relevant prior research that uses a theoretically similar manipulation with the same dependent variable. Maybe a previous similar experiment found a difference of 0.85 units. This helps inform us, but of course one cannot generalize perfectly from past experiments. So based on the principle "when in doubt spread it out," round up to 1 unit and use this as the mean of a two tailed normal with an *SD* equal to half that value. See Figure A3.
4. Alternatively if one thought smaller values were more likely than larger values, take the (rounded up) value from past research and use it as the standard deviation of a half normal with a mode of zero. See Figure A4.
5. It may be difficult to find past research with the same dependent variable. The best way of scaling predictions may be with a standardized effect size (Cohen's *d* for between-subjects and *dz* for within-subjects, or the correlation *r* for either). One could use an estimate of the relevant *SD* in the dependent variable one is using (within-group *SD* for *d* and *SD* of differences for *dz*) to convert the standardized effect to a raw difference and use Guideline 3 or 4 above. Or more elegantly, directly use the standardized effect size, Cohen's *d* or *dz,*[12] to scale the predictions of the Rouder Bayes factor calculator (http://pcl.missouri.edu/bayesfactor). Bear in mind that the predictions in the Rouder calculator are treated as two tailed, so the Bayes factor is not appropriate for theories that predict a direction. Alternatively, the standardized effect size can be represented as a correlation, *r*, which can be converted a normally distributed variable, allowing the Dienes calculator to be used to flexibly represent different theories (e.g., directional ones). I now consider an example.

Wetzels et al. (2011) consider an experiment by Mussweiler (2006), who assessed whether unobtrusively inducing people to move in a portly manner would (in comparison with a control group) make subjects view an ambiguous target person as more overweight. The significance test yielded $t(18) = 2.1$, $p = .05$. Wetzel

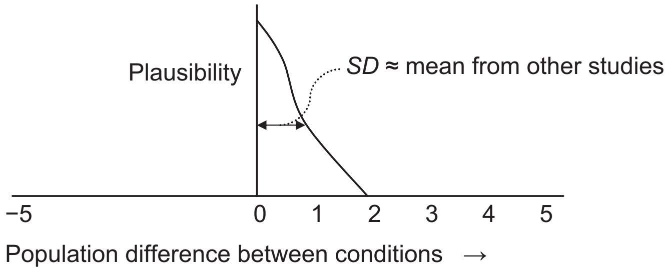

Fig. A4.

et al. use the Rouder Bayes factor, which assumes an alternative theory for which effects in either direction are just as plausible, and scaled for large effects (Cohen's $d = 1$). The Bayes factor was 1.56, barely evidence for the Rouder alternative theory over the null. However, Mussweiler clearly makes a directional prediction so the theory is not directly assessed by the Rouder Bayes factor. I will use the Dienes calculator to obtain a Bayes factor that assesses Mussweiler's theory.

To convert Mussweiler's obtained effect into a correlation coefficient *r*, use the formula: $r^2 = t^2 / (t^2 + df)$. Thus, the obtained effect is $r = \sqrt{[2.1^2(2.1^2 + 18)]} = 0.44$. *r* can be converted to Fisher's *z* to make it normally distributed: Fisher's $z = 0.5 \times \log_e[|(1 + r) / (1 - r)|] = 0.5 \times \log_e (1.44 / 0.56) = 0.47$. Fisher's *z* has a standard error of $1 / \sqrt{(N-3)}$ where *N* is the total number of subjects. So standard error $= 1 / \sqrt{(20 - 3)} = 0.24$. So far, we have found the summary of the data needed to enter into the Dienes Bayes factor calculator: The mean is .47 and the standard error is .24.

Now we need to specify the predictions of the theory. Mussweiler bases his effect on previous similar social priming explored by Bargh, Chen, and Burrows (1996), who found large effects (Cohen's *d* of about one). We will use Guideline 4 above, and scale with a large effect size. A Cohen's *d* of 1 corresponds to an *r* of .44,[13] which is a Fisher's *z* of .47. So to represent the predictions of the theory, we will use a half-normal distribution with a mode of zero and a standard deviation of .47. This gives a Bayes factor of 4.00, substantial evidence for Mussweiler's theory over the null. The example shows the difference between using an automatic Bayes factor (as Wetzel

et al. do, Bayes factor = 1.54), and a Bayes factor designed to assess a theory an author has in mind (as we have done here, Bayes factor = 4).

When you assess a theory by raw effect size, it makes you think clearly about the absolute magnitude of the effect; when you assess a theory with standardized effect size, the concern is with the consistency of the effect. Both can be relevant, but bear in mind that standardized effects will be affected by considerations unrelated to most theories, such as number of trials and other factors in the analysis that reduce mean square error. Thus, for the most part, theories will be best assessed by considering their predictions in terms of a raw effect size on a meaningful scale.

Declaration of Conflicting Interests

The author declared that he had no conflicts of interest with respect to his authorship or the publication of this article.

Notes

1. Power can be calculated in the Bayesian approach to determine likely numbers of subjects needed to make a point, though this is a practical matter, and power does not figure in the inferential procedure itself, unlike in the Neyman Pearson approach (see Kruschke, 2010 a, 2010b, 2010c; Royall, 1997).
2. Bayes theorem is a means for converting one conditional probability, for example, $P(D \mid H)$, into its inverse, $P(H \mid D)$. It states that $P(H \mid D) = P(D \mid H) \times P(H) / P(D)$. The expression in the text ignores $P(D)$, the probability of the data, because $P(D)$ is not needed when the data is treated as fixed rather than variable: $P(D)$ either just becomes a scaling constant for making $P(H \mid D)$ sum to unity (over the distribution of possible hypotheses) or it cancels out, as in the Bayes factor below. Note that this is a key difference with significance testing: To get a p value, the hypothesis is fixed and different possible data are considered; in Bayesian statistics, the data are given and different possible hypotheses are considered.
3. Using the Bayes rule for two hypotheses, *H1* and *H2*, for given data, *D*, we have
   $P(H1 \mid D) = P(D \mid H1) \times P(H1) / P(D)$
   and
   $P(H2 \mid D) = P(D \mid H2) \times P(H2) / P(D)$.
   When dividing the former by the latter
   $P(H1 \mid D) / P(H2 \mid D) = (D \mid H1) / P(D \mid H2) \times P(H1) / P(H2) \times P(D) / P(D)$,
   the last term cancels out and we are left with
   ratio of posterior probabilities = ratio of likelihoods × ratio of priors (i.e., Posterior odds = Bayes factor * prior odds).
4. Kruschke (2010a) considers another stopping rule similar to one people often use: Collect as many subjects as you can until the end of the week (or as many students as turn up for the practical, etc.). Different applications of this rule will produce differing numbers of subjects. Thus, to calculate a p value, one cannot just use the value from SPSS, which implicitly assumes the stopping rule was to run until reaching the precise number of subjects obtained. Instead, one needs to take into account the distribution of the different numbers of subjects the rule entails one might have run; one could run a simulation of thousands of experiments to determine the resulting distribution of t and, hence, the p value. Simply using the output of SPSS is cheating.
5. Consider 20 independent tests of ESP on different psychics; each psychic has to select the right item out of 20 items. So P(correct answer | H_0) = .05 and P(incorrect answer | H_0) = .95 for the test for each psychic. Let us assume P(correct answer | psychic has ESP) = .95 and P(incorrect answer | psychic has ESP) = .05. Thus, if only one psychic passes the test out of 20, the likelihood of the theory that all psychics have ESP is $.95(.05)^{19}$ and the likelihood of the family-wise null is $(0.95)^{19}.05$. Thus, the Bayes factor in favor of the theory that all the psychics have ESP over the null is $(1/19)^{18}$. Further, the Bayes factor for the theory that there is a 1/20 probability that any one of them has ESP, over the null, is 0.75. The data support the family-wise null over either of these theories that ESP exists.
6. As Halpern (1999) indicates, Cox did assume that beliefs are strictly continuous (i.e., that one can make infinitesimally small distinctions between different degrees of belief). Of course, this is not realistic for our actual beliefs; nonetheless, Cox's degrees of belief can still serve as normative models.
7. One must specify m, the minimal effect that could plausibly be expected given the theory. Then, assuming that any population effect is due to the theory, the null can be accepted if a nonsignificant result was obtained and there was sufficient power to detect m or if the confidence interval includes zero but excludes m (see Dienes, 2008). Thus, specifying the power for a "medium effect size" is only inferentially relevant if a medium effect happens to be the minimal effect that could plausibly be expected given the theory.
8. Crediblity intervals can be used when one believes any precise value, including zero, has zero probability; if one wanted to give a finite probability to a precise value, like a difference of precisely zero, then Bayes factors are appropriate. For a standard credibility interval, the null hypothesis has zero probability before and after collecting data, so it can always be rejected with complete confidence.
9. Consider the following example. The data are passing or failing a test—if a person fails they get a 0, if they pass they get a 1. You should be able to see that the distribution of scores is not at all normal in any way. What is the population mean? If 40% of people pass, the mean is .4. If 70% pass, the mean is .7. I might have reason to think (e.g., based on past papers) that about 60% of people pass and that values more than this are increasingly unlikely and values less than this are increasingly unlikely. So I could represent the plausibility of different possible mean values as a normal with a mean of 0.6 and, say, a standard deviation of 0.1. This indicates that I am pretty sure the population mean lies between 0.4 and 0.8. But it is important to note that no subject has a score between 0.4 and 0.8. Each subject is either 0 or 1. Just so, the distributions we have been talking about are the distribution of the plausibility of population mean values and not the distribution of the data.
10. To get these results, enter the following into the Bayes factor calculator: Answer "no" to the question "Is the distribution uniform?"; enter "0" for the mean of the distribution, "1" for the standard deviation, and "1" for number of tails. These entries

define the predictions of the theory. Now for the data. When the mean is one standard error in the right direction, enter "1" for mean and "1" for standard error; when the mean is 0, enter "0" for mean and '"1" for standard error; when the mean is one standard error in the wrong direction, enter "−1" for the mean and "1" for standard error.

11. For example, according to signal detection theory, Type II *d*' cannot be greater than Type I (so this can be a constraint in investigating metacognition; see Dienes, 2010).
12. For a between-group *t* test, Cohen's $d =$ (mean difference between groups) / (pooled *SD* within a group). If there are equal numbers of subjects (n) within each group, then $d = t\sqrt{(2/n)}$. For a within-subjects or one-sample *t* test, Cohen's $dz =$ (mean difference)/(standard deviation of the difference scores). To obtain *dz* from a paper, use $dz = t/\sqrt{n}$.
13. $r^2 = d^2/(d^2 + 4)$ for the between subjects case, and $r^2 = dz^2/(dz^2 + 1)$ for the within-subjects case.

References

Armitage, P., Berry, G., & Matthews, J.N.S. (2002). *Statistical methods in medical research* (4th ed.). Malden, MA: Blackwell.

Armitage, P., McPherson, C.K., & Rowe, B.C. (1969). Repeated significance tests on accumulating data. *Journal of the Royal Statistical Society: Series A. Statistics in Society*, *132*, 235–244.

Baguley, T. (2009). Standardized or simple effect size: What should be reported? *British Journal of Psychology*, *100*, 603–617.

Baguley, T. (in press). *Beyond analysis of variance*. Hampshire, England: Palgrave Macmillian.

Baguley, T., & Kaye, W.S. (2010). Review of "Understanding Psychology as a Science: An Introduction to Scientific and Statistical Inference". *British Journal of Mathematical & Statistical Psychology*, *63*, 695–698.

Bargh, J.A., Chen, M., & Burrows, L. (1996). Automaticity of social behavior: Direct effects of trait construct and stereotype activation on action. *Journal of Personality and Social Psychology*, *71*, 230–244.

Berger, J. (2003). Could Fisher, Jeffreys and Neyman have agreed on testing? *Statistical Science*, *18*, 1–32.

Birnbaum, A. (1962). On the foundations of statistical inference. *Journal of the American Statistical Association*, *53*, 259–326.

Blume, J. D. (in press). Likelihood and its evidential framework. In P.S. Bandyopadhyay & M. Forster (Eds.), *Handbook of the philosophy of statistics*. Amsterdam: Elsevier.

Coulson, M., Healey, M., Fidler, F., & Cumming, G. (2010). Confidence intervals permit, but do not guarantee, better inference than statistical significance testing. *Frontiers in Psychology*, *1*, article 26. Retrieved from http://www.frontiersin.org/quantitative_psychology_and_measurement/10.3389/fpsyg.2010.00026/full

Cox, R.T. (1946). Probability, frequency, and reasonable expectation. *American Journal of Physics*, *14*, 1–13.

Dienes, Z. (2008). *Understanding psychology as a science: An introduction to scientific and statistical inference*. Hampshire, England: Palgrave Macmillan. Retrieved from http://www.lifesci.sussex.ac.uk/home/Zoltan_Dienes/inference/

Dienes, Z. (2010). Measuring the conscious status of knowledge. In L.A.P. Miranda & A.I. Madariaga (Eds.), *Advances in cognitive science: Learning, evolution and social action* (pp. 113–128). Bilbao, Spain: University of the Basque Country Press.

Fidler, F., Thomason, N., Cumming, G., Finch, S., & Leeman, J. (2004). Editors can lead researchers to confidence intervals, but can't make them think. *Psychological Science*, *15*, 119–126.

Gigerenzer, G. (2004). Mindless statistics. *Journal of Socio-Economics*, *33*, 587–606.

Halpern, J.Y. (1999). Cox's theorem revisited. *Journal of Artificial Intelligence Research*, *11*, 429–435.

Harlow, L.L., Mulaik, S.A., Steiger, J.H. (Eds.). (1997). *What if there were no significance tests?* Mahwah, NJ: Erlbaum.

Hoijtink, H., Klugkist, I., & Boelen, P.A. (Eds.). (2008). *Bayesian evaluation of informative hypotheses*. New York: Springer.

Howard, G.S., Maxwell, S.E., & Fleming, K.J. (2000). The proof of the pudding: An illustration of the relative strengths of null hypothesis, meta-analysis, and Bayesian analysis. *Psychological Methods*, *5*, 315–332.

Howson, C., & Urbach, P. (2006*). Scientific reasoning: The Bayesian approach* (3rd ed.). Chicago: Open Court.

Jaynes, E.T. (2003). *Probability theory: The logic of science*. Cambridge, England: Cambridge University Press.

Jeffreys, H. (1961). *The theory of probability* (3rd ed.). Oxford, England: Oxford University Press.

Johansson, T. (in press). Hail the impossible: *p* values, evidence, and likelihood. *Scandinavian Journal of Psychology*.

Kerr, N.L. (1998). HARKing: Hypothesizing after the results are known. *Personality and Social Psychology Review*, *2*, 196–217.

Kruschke, J.K. (2010a). Bayesian data analysis. *Wiley Interdisciplinary Reviews: Cognitive Science*, *1*, 658–676.

Kruschke, J.K. (2010b). *Doing Bayesian data analysis: A tutorial with R and BUGS*. Burlington, MA: Academic Press.

Kruschke, J.K. (2010c). What to believe: Bayesian methods for data analysis. *Trends in Cognitive Sciences*, *14*, 293–300.

Kruschke, J.K. (2011). Bayesian assessment of null values via parameter estimation and model comparison. *Perspectives on Psychological Science*, *6*, 299–312.

Lakatos. I. (1978). *The methodology of scientific research programmes: Philosophical papers* (Vol. 1). Cambridge, England: Cambridge University Press.

Lakatos, I., & Feyerabend, P. (1999). *For and against method*. Chicago: University of Chicago Press.

Mayo, D. (1996). *Error and the growth of experimental knowledge*. Chicago: University of Chicago Press.

Mayo, D.G. (2004). An error statistical philosophy of evidence. In M.L. Taper & S.R. Lele (Eds.), *The nature of scientific evidence: Statistical, philosophical and empirical considerations* (pp. 79– 96). Chicago: University of Chicago Press.

Meehl, P. (1967). Theory-testing in psychology and physics: A methodological paradox. *Philosophy of Science*, *34*, 103–115.

Miller, D. (1994). *Critical rationalism: A restatement and defence*. Chicago: Open Court.

Mussweiler, T. (2006). Doing is for thinking! *Psychological Science*, *17*, 17–21.

Oakes, M. (1986). *Statistical inference: A commentary for the social and behavioural sciences*. Hoboken, NJ: Wiley.

Popper, K. (1963). *Conjectures and refutations*. London: Routledge.

Rouder, J.N., Morey, R.D., Speckman, P.L., & Pratte, M.S. (2007). Detecting chance: A solution to the null sensitivity problem in subliminal priming. *Psychonomic Bulletin & Review*, *14*, 597–605.

Rouder, J.N., Speckman, P.L., Sun, D., Morey, R.D., & Iverson, G. (2009). Bayesian *t* tests for accepting and rejecting the null hypothesis. *Psychonomic Bulletin & Review*, *16*, 225–237.

Royall, R.M. (1997). *Statistical evidence: A likelihood paradigm*. London: Chapman & Hall.

Savage, L.J. (1962). *The foundations of statistical inference: A discussion*. London: Methuen.

Sivia, D.S., & Skilling, J. (2006). *Data analysis: A Bayesian tutorial* (2nd ed.). Oxford, England: Oxford University Press.

Taper, M.L., & Lele, S.R. (2004). *The nature of scientific evidence: Statistical, philosophical and empirical considerations*. Chicago: University of Chicago Press.

von Mises, R. (1957). *Probability, statistics and truth*. London: Macmillan.

Wagenmakers, E.J. (2007). A practical solution to the pervasive problems of *p* values.*Psychonomic Bulletin & Review*, *14*, 779–804.

Westfall, P.H., Johnson, W.O., & Utts, J.M. (1997). A Bayesian perspective on the Bonferroni adjustment. *Biometrika*, *84*, 419–427.

Wetzels, R., Matzke, D., Lee, M.D., Rouder, J.N., Iverson, G.J., & Wagenmakers, E.J. (2011). Statistical evidence in experimental psychology: An empirical comparison using 855 *t* tests. *Perspectives on Psychological Science*, *6*, 291–298.

Wright, D.B. (2010). Ten statisticians and their impacts for psychologists. *Perspectives on Psychological Science*, *4*, 587–597.

Ziliak, S.T., & McCloskey, D.N. (2008). *The cult of statistical significance: How the standard error cost us jobs, justice and lives*. Ann Arbor: University of Michigan Press.
